# Single-cell transcriptomics delineates the immune cell landscape in equine lower airways and reveals upregulation of FKBP5 in horses with asthma

**DOI:** 10.1038/s41598-023-43368-4

**Published:** 2023-09-27

**Authors:** Miia Riihimäki, Kim Fegraeus, Jessica Nordlund, Ida Waern, Sara Wernersson, Srinivas Akula, Lars Hellman, Amanda Raine

**Affiliations:** 1 Department of Clinical Sciences, Swedish University of Agricultural Sciences, Uppsala, Sweden; 2 Department of Medical Sciences, Science for Life Laboratory, Uppsala University, Uppsala, Sweden; 3 Department of Anatomy, Physiology and Biochemistry, Swedish University of Agricultural Sciences, Uppsala, Sweden; 4 Department of Cell and Molecular Biology, Uppsala University, Uppsala, Sweden

**Keywords:** Gene expression, Molecular medicine, Asthma

## Abstract

Equine asthma (EA) is a heterogenous, complex disease, with a significant negative impact on horse welfare and performance. EA and human asthma share fundamental similarities, making EA a useful model for studying the disease. One relevant sample type for investigating chronic lung inflammation is bronchoalveolar lavage fluid (BALF), which provides a snapshot of the immune cells present in the alveolar space. To investigate the immune cell landscape of the respiratory tract in horses with mild-to-moderate equine asthma (mEA) and healthy controls, single-cell RNA sequencing was conducted on equine BALF cells. We characterized the major immune cell populations present in equine BALF, as well as subtypes thereof. Interestingly, the most significantly upregulated gene discovered in cases of mEA was FKBP5, a chaperone protein involved in regulating the activity of the glucocorticoid receptor.

## Introduction

Asthma is one of the most prevalent chronic diseases impacting the airways of both humans and specific domestic animals. In horses, equine asthma (EA) represents a significant welfare concern, standing as one of the primary contributors to compromised performance among athletic equines. Similar to its human counterpart, EA is a heterogeneous, complex disease associated with both genetic and environmental factors^1,2^. Two of the main environmental factors influencing the prevalence and severity of the disease are stable conditions and feed quality^2,3^, which are both related to the exposure to dust and mold. The clinical symptoms of EA are similar to human asthma and include coughing, airway obstruction, increased respiratory effort and mucus accumulation, as well as decreased athletic performance^1,4–6^.

Presently, EA is categorized into two main subtypes: severe equine asthma (sEA), formerly recognized as recurrent airway obstruction (RAO), and mild-moderate equine asthma (mEA), previously referred to as inflammatory airway disease (IAD)^1,7^. This classification into severe and mild-to-moderate variants of the disorder is employed for clinical case differentiation and is based on both symptom severity and the presence and frequency of specific inflammatory cell types within bronchoalveolar lavage (BALF)^8^. sEA usually displays clear characteristics such as increased respiratory effort along with a high degree of neutrophilic inflammation. On the other hand, mEA is more heterogenous in terms of symptoms and inflammatory cell compositions in BALF^9^. It is likely that additional endotypes exists under the EA umbrella term and that the division into mEA and sEA in horses is too coarse^10^.

Given that horses are among the few animals that can spontaneously develop asthma, sEA has been proposed as a valuable animal model for human neutrophilic asthma. Moreover, similarities in features have been identified between sEA and both allergic and non-allergic human asthma phenotypes^1^. However, while shared features between mild equine asthma (mEA) and those human asthma phenotypes have also been identified^1^, mEA displays greater diversity than sEA. Therefore, the extent to which mEA can effectively serve as a model for various subtypes of human asthma remains unclear.

EA is traditionally diagnosed based on medical history, clinical examination, airway endoscopy findings, lung function tests, and BALF cytology^8^. Increased granulocyte infiltration in the lungs of horses affected with both sEA and mEA makes BALF cytology a valuable tool for diagnosing the disease^11,12^. Traditional BALF cytology, however, does not provide sufficient resolution to study disease mechanisms or perform high-resolution subclassification of asthma. High throughput RNA sequencing can be valuable to obtain more detailed information based on gene expression and have been used in several studies to study gene expression differences in BALF cells and bronchial biopsies sampled from horses diagnosed with asthma^13–17^. Traditional bulk RNA-sequencing technologies obtain measurements from a mixture of cells in which intercellular differences are averaged. In contrast, single cell RNA sequencing (scRNA-seq) is a technology that offers possibilities to analyze gene expression at the individual cell level, which enables characterization of cellular diversity. Although many studies have been conducted with cells of human and mouse origin, very few studies have been published using horse scRNA-seq^18–20^. To our knowledge, there is yet no comprehensive atlas of the equine BALF cell landscape including cells from both mEA cases and healthy controls^19,20^. In the present study, detailed information on the cell types occupying the alveolar space of horses in health and disease was obtained by conducting scRNA-seq on clinically relevant BALF samples. Furthermore, significantly differentially expressed (DE) gene transcripts were observed in the BALF cells from horses with mEA, which can be further explored as potential biomarker for the disease and for prediction of therapy response in horse and human.

## Results

### The global cellular landscape in the lower airways of horses in health and disease as detected by scRNA-seq

A total of nineteen horses were included in the study. Eleven horses were client-owned and presented at the veterinary clinic for asthma evaluation, while eight control horses were sampled from the research herd at the Swedish University of Agricultural Sciences (SLU). A complete description of cases and controls, clinical scoring and inclusion criteria is provided in the Methods section and in Supplementary Information S1 (Study Design).

As part of routine diagnostic procedures, the cellular compositions of BALF samples were evaluated through cytology staining (Table 1). Among the mEA-affected horses, there was a notable increase in mast cell counts (4–11%) compared to the healthy controls (p = 0.0005, Kruskal–Wallis). Additionally, two mEA horses exhibited elevated neutrophil levels, whereas six horses demonstrated heightened eosinophil counts in comparison to the control group (0–18%, p = 0.007, Kruskal–Wallis). As a result, the mEA cases included horses primarily characterized by a mastocytic phenotype, along with phenotypes featuring mixed granulocytes. Horse FN in the mEA group did not meet the inclusion criteria for mEA as it displayed normal BALF counts (and was thus excluded from statistical tests). The control horses exhibited normal BALF cell counts, except for horse B, which, due to elevated neutrophil counts, did not meet the inclusion criteria for the controls.

**Table 1:** Total BALF cell counts, BALF cytology data and number of cells analyzed with DropSeq, per horse.

ID	Pheno-type	Leukocytes (10e6/L)	Neutrophils (%)	Lymphocytes (%)	Macrophages (%)	Eosinophils (%)	Mast cells (%)	No of cells*
MW	Asthma	320	16	48	28	3	5	3367
O	Asthma	400	4	22	49	18	7	5224
Z	Asthma	550	18	28	45	5	4	2716
N	Asthma	310	0	37	47	6	10	3421
FS	Asthma	190	7	33	42	8	10	3003
C	Asthma	460	2	41	50	0	7	2709
VE	Asthma	< 100	3	37	52	1	7	4461
VA	Asthma	280	5	12	71	6	6	3060
T	Asthma	190	5	33	53	0	9	3393
D	Asthma	180	1	35	52	1	11	3092
FN	n.d	140	1	36	61	0	2	2067
MY	Control	350	3	46	50	0	1	3885
A	Control	250	7	30	62	0	1	2732
B	n.d	470	13	36	49	1	1	3326
F	Control	250	3	44	51	0	2	3292
H	Control	510	5	44	50	0	1	3625
Q	Control	150	6	29	64	0	1	3437
G	Control	200	3	31	63	1	2	4207
P	Control	170	4	30	65	0	1	3705

nd = phenotype not defined. FN = clinical patient with normal BALF, B = control horse with neutrophils in BALF, both excluded from DE analysis.

*Nr of single cell transcriptomes recovered per horse after quality filtering.

**Figure 1:**
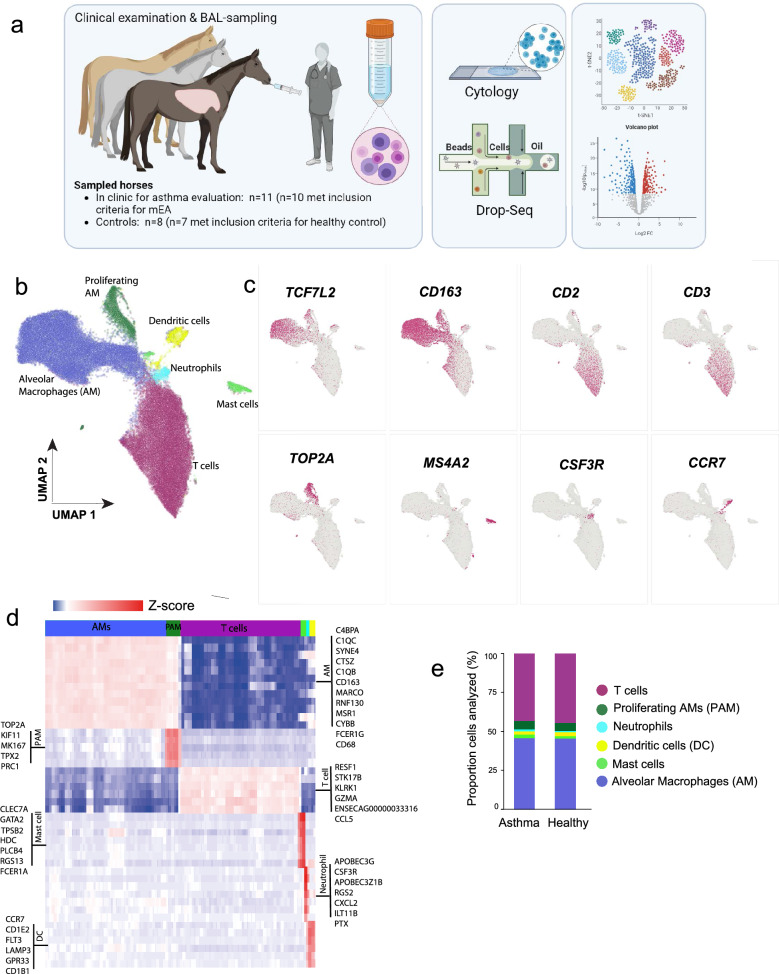
Initial clustering of BALF cells (**a**) Graphical illustration of the study design. The figure was created using the BioRender software. (**b**) UMAP representation of 63,022 equine BALF cells colored by the six major cell groups: alveolar macrophages (28,277 cells), proliferating macrophages (3420 cells), T cells (28,112 cells), mast cells (1257 cells), neutrophils (797 cells) and dendritic cells (1339 cells). (**c**) Example of expression of specific markers for the different cell types. (**d**) Heat map demonstrating the difference in expression levels of the top biomarker genes for each of the six major clusters. (**e**) Proportion of cell-types in the EA and healthy control horses, by group.

Single cell RNA sequencing of cells in BALF from the nineteen horses sampled in the study was performed using the Drop-Seq method (as outlined in Fig. 1a)^21^. After single cell encapsulation, sequencing and filtering of data using stringent quality thresholds (see Methods and Supplementary Fig. S2), the single cell transcriptomes from in total 63,022 cells remained for integrated clustering and cell type annotation (mEA n = 36,513 cells, healthy n = 28,209 cells). A total of 11,844 genes were detected. Principal component analysis (PCA) showed a weak tendency for clustering according to health status, but there was no clear separation between the asthma and control samples (Supplementary Fig. S2).

Clustering of the major cell populations in BALF were visualized by UMAP (Fig. 1b, Supplementary Table S2 for a complete list of cluster markers)^22^. As expected, the most abundant immune cell types were alveolar macrophages (AMs) and T cells. AMs were identified based on the expression of established canonical macrophage markers: *MARCO*, *CD163*, *CD68*. Moreover, the Wnt effector transcription factor *TCF7L2* was highly expressed in equine AMs^23,24^ (Fig. 1c,d; Supplementary Table S2). A substantial population (3077 cells) of proliferating AMs was identified. In addition to canonical macrophage markers genes, this population specifically expressed differentiation and cell division genes, such as *TOP2A*, *KIF11*, *MK167* and *TPX2*.

T cells were annotated based on expression of classical surface receptor T cell markers (*CD*2, *CD3*), cytotoxicity genes (*GZMA*, *KLRK1*), chemokine (*CCL5*), T cell receptor (*TRCB1*/*ENSECAG00000033316*), and the serine/threonine kinase *STK17B* (Fig. 1c,d).

A notable subgroup of cells (n = 2800) was observed, clustering with T cells and concurrently exhibiting co-expression of canonical macrophage markers (double positive cells) (Supplementary Fig. S2). T cell/monocyte double positive (DP) cells have been observed previously, e.g., in blood^25,26^. It is likely that the DP cells observed herein represent either technical doublets (where two or more cells enter the same droplet by chance) and/or captures functional T cell/AM complexes in the same droplet. The DP cells were excluded from the downstream analysis.

Mast cells were annotated according to expression of tryptase *TPSB2*, the high affinity IgE receptor beta and alpha chains (*MS4A2*, *FCER1A*) and the transcription factor *GATA2.* A small cluster of neutrophils (Fig. 1b, light blue cluster) were annotated according to expression of e.g., the chemokine *CXCL2*, the granulocyte colony stimulating factor *CSF3R*, *APOBEC*-family transcripts and *CD85* (*ILT11B*). Another small cluster (Fig. 1b, yellow cluster) were characterized by high expression of *CD1*, *CCR7*, *LAMP3* and *FLT3*, which are known markers for dendritic cells^27–30^.

When comparing the scRNA-seq data to the routine cytology analysis, the majority of cells detected were AMs and T cells, as expected (Fig. 1; Table 1). The proportions of granulocytes were lower in the scRNA-seq data compared to the cytology analysis. Notably, the clustering did not confidently designate an eosinophil cluster, despite the elevated eosinophil BALF cytology counts observed for a subset of the EA horses included in the study. Although there was a clear difference in mast cell counts (from cytology) between the EA and healthy controls (Table 1), comparison of the proportions of the various cell types between the asthma and healthy group, as identified by scRNA-seq, showed no major difference between the groups (Fig. 1e, Supplementary Fig. S3, Supplementary Table S3).

### Distinct subgroups of equine T cells populate the alveolar compartment of horses

**Figure 2:**
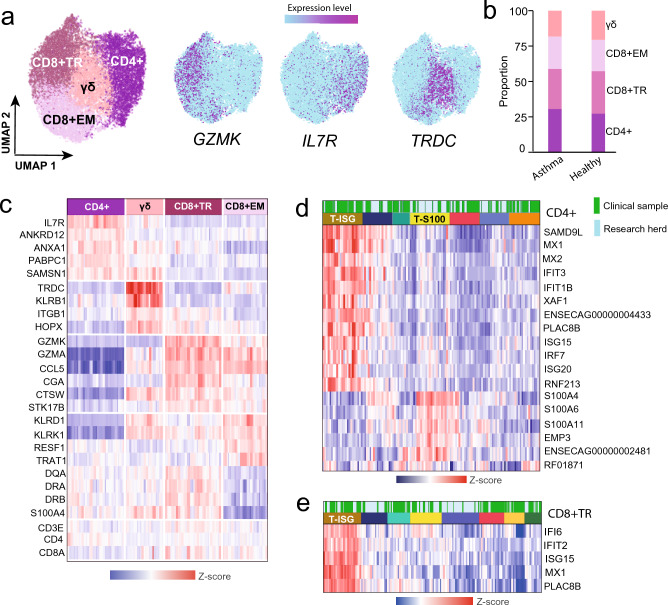
Re-clustering of T cells. (**a**) The UMAP plot illustrates the four equine BALF T cell groups. The expression of three top differentially expressed markers is mapped onto the UMAP plot, which is shown to the right. (**b**) The proportion of T cell subtypes in the EA vs healthy group of horses is depicted. (**c**) The differential expression of T cell cluster markers visualized with a heat map. (**d**) Iterative clustering reveals seven subpopulations of CD4^+^ cells. The subset coloured in brown in the heat map (T-ISG^Hi^) expresses interferon stimulated genes, and the yellow subset (T-S100^Hi^) displays higher expression of S100 genes and other genes involved in cytoskeleton organization and cell adhesion, as indicated by GO analysis. The heat map shows the genes with the highest fold changes in the T-ISG^Hi^ and T-S100^Hi^ relative to the other clusters. The green label indicates data from the horses sampled at the clinic and the blue label indicates data from the research herd. (**e**) Iterative clustering of the CD8^+^_TR_ cluster shows eight subclusters. The brown subset (T-ISG^Hi^) selectively expresses interferon stimulated genes. The heat map shows the genes with the highest fold changes in the T-ISG^Hi^ cluster relative to the other clusters. See Supplementary Fig. S3 and Supplementary Table S3 for the proportions of case and control cells within the clusters.

In order to increase the cluster resolution and to facilitate annotation of different T cell populations, the 23,480 T cells were independently sub-clustered (excluding the putative T cell-macrophage DP cells). This initially resulted in six T cell clusters (Supplementary Fig. S4), where four contained clear signatures based on previously defined T cell phenotype (Fig. 2a,b; Supplementary Fig. S4). Focusing on the cells in those four T cell clusters, two clusters were annotated as CD8^+^ T cells (hereafter denoted CD8^+^_TR_ and CD8^+^_EM_), one cluster as CD4^+^ T cells, and one cluster as γδ-T cells. The four major T cell types were found in all horses, and no significant differences in the proportions between the two conditions were observed (Fig. 2b; Supplementary Fig. S3, p-values > 0.05, Supplementary Table S3, Kruskal–Wallis test).

The cells in the CD8^+^ clusters expressed genes associated with cytotoxicity as well as known CD8^+^ T cell markers (Fig. 2a,c). In total, 48 differentially expressed genes (log2ratio > 1, fold change > 2) were identified when comparing the two CD8^+^ T cell clusters (Fig. 2c; Supplementary Fig S4; Supplementary Table S4). Gene ontology (GO) pathway analysis using the set of genes with > twofold higher expression in the CD8^+^_TR_ cluster indicated enrichment to biological pathways involved in adhesion, migration, and T cell activation (Supplementary Fig. S4). The CD8^+^_TR_ cluster expressed higher levels of transcripts typical of cytotoxic CD8^+^ T cells, such as granzymes (*GZMA*, *GZMK*), the cytotoxicity associated cathepsin W (*CTSW*), killer lectin (*KLRK1*) and chemokines (*CCL5*)*.* In the same cluster, a higher transcription of integrins and other genes implicated in adhesion and migration (*IGTAE*, *ITGB*, *S100A4*, *S100A11*, and *ANXA1*) was also observed, compared to the CD8^+^_EM_ cluster.

The expression of MHC class II genes (*DQA*, *DQB*, *DRA*, and *DRB*) was also elevated in the CD8^+^_TR_ cluster. Interestingly, higher expression of the *CGA* gene (the alpha chain of glycoprotein hormones) was detected in this cluster. To our knowledge, this gene has not previously been described in T cell function. Taken together, the expression signature of the CD8^+^_TR_ cluster suggests a population of active tissue resident CD8^+^ memory cells.

The CD8^+^_EM_ cluster was also characterized by high *GZMA*, *GZMK* and *CCL5* expression, albeit lower *GZMK* expression than the CD8^+^_TR_ cluster. Moreover, expression of *KLRD1*, *RESF1* and *TRAT1* were higher in this cluster (Fig. 2c; Supplementary Fig. S4). The lower levels of CD103 (*ITGAE*) and other adhesion-related transcripts in this cluster indicate a population of circulating effector memory T cells (CD8^+^_EM_)^31^.

Compared to the other T cell clusters, the CD4^+^ cluster was characterized by upregulation of *IL7R*, *ANXA1*, *PABPC1*, *TNFSF13B*, and *SAMSN1*. A distinct subset of CD4^+^ cells displayed higher expression of S100 family genes (*S100A4*, *S100A6* and *S100A11*) as well as *EMP3* (Epithelial membrane protein 3), a protein thought to be involved in cell proliferation and cell–cell interactions. Additionally, GO analysis revealed upregulation of pathways related to cytoskeleton organization in this population (Supplementary Table S4 and Supplementary Fig. S5). Notably, this cluster was found to mostly comprise T cells derived from the healthy control horses (p-value = 0.002, Fig. 2d, Supplementary Fig. S3 and Supplementary Table S3). However, the CD4^+^ cluster could not be subclassified into any of the conventional Th-subtypes (Th1, 2, 17, T-reg) based on the single cell expression signatures obtained in this study.

Of note, subsets of cells were identified within the CD4^+^ and CD8^+^_TR_ clusters, showing a significant higher expression of known interferon stimulated genes (ISG)^32^, including *MX1*, *PLAC8D*, *IFIT3*, and *ISG15* (Fig. 2d,e; Supplementary Fig. S4; Supplementary Table S4). Upon further iterative clustering of the CD4^+^ and CD8^+^_TR_ populations, the ISG expressing T cells (T-ISG^Hi^) fell into distinct sub-clusters (Fig. 2d,e). While the T-ISG^Hi^ cells were found in the majority of horses, a trend was observed towards higher proportions of these cells in EA (2.8-fold increase, p-value = 0.10 in the CD4^+^ cells) (Supplementary Fig. S3, Supplementary Table S3). The T cells and macrophages from two horses (N and C) in the EA group exhibited more pronounced expression of a number of ISG genes, compared to the other horses (Supplementary Fig. S6).

The γδ-T cells were characterized by high T cell receptor delta constant *TRDC*, lower alpha constant *TRAC* and high *CTSW.* Moreover, the γδ-T cell population exhibited a high level of transcripts for the killer lectin receptor *KLRB1* and also expressed *KLRD1* and *KLRK1* (Fig. 2; Supplementary Fig. S4, Supplementary Table S4)*.* Altogether, this suggests cytotoxic potential and is in agreement with what was demonstrated for circulating γδ-T cells in a previous scRNA-seq study of the equine PBMC fraction^18,20^.

Although the two remaining clusters (T_NA_ and T_DP_) expressed known T cell markers, no specific markers were detected that could enable further annotation of those clusters to a specific T cell subtype (Supplementary Fig. S4).

Through iterative clustering of a large population of T cells, the major subtypes present in the equine respiratory tract were delineated, revealing novel equine T cell populations characterized by higher expression of interferon-stimulated genes (T ISG^Hi^) and S100 proteins (T S100^Hi^).

### Heterogeneity among equine alveolar macrophages

**Figure 3:**
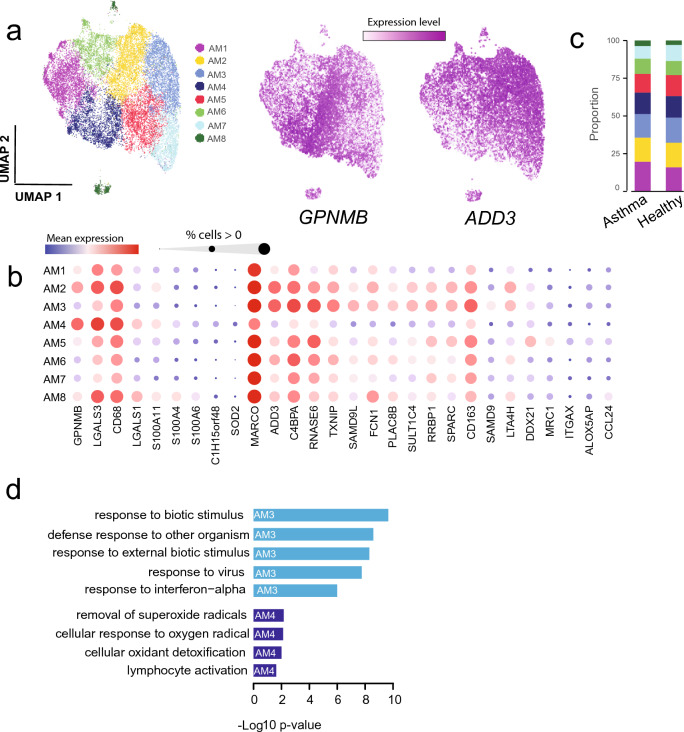
Re-clustering of alveolar macrophages. (**a**) UMAP illustration of eight alveolar macrophage (AM) subsets. The top differentially expressed markers for the AM4 cluster and AM3 cluster are glycoprotein Nmb (*GPNMB*) and γ-adductin (*ADD3*), respectively. (**b**) Bubble plot visualizing the expression levels for genes which were among the top differentially expressed between AM clusters. (**c**) Proportion of AM clusters in the EA and healthy groups. (**d**) Genes upregulated in cluster AM3 were significantly enriched in the GO pathways colored in light blue. Genes upregulated in cluster AM4 were significantly enriched in GO pathways colored in dark blue.

In a similar manner as for the T cells, re-clustering of AMs was performed (excluding the cluster of proliferating AMs). As AMs are large cells with relatively high RNA content, we applied the stringent cut-off of including only cells expressing > 500 genes. This resulted in 22,000 AM cells for downstream iterative clustering (Fig. 3a). Given that recent studies have demonstrated considerable heterogeneity among human and mouse AMs, high diversity is to be expected also for horse AMs^33,34^. Sub-clustering revealed several equine AM subpopulations with distinct gene expression profiles (Fig. 3a). Same as for the T cell sub-populations, no pronounced difference in the proportions was found when comparing EA and healthy controls (Fig. 3c; Supplementary Fig. S3; Supplementary Table S3). Within the AMs, the cluster denoted AM4 had the largest number of expression differences compared to the other clusters (> 300 differentially expressed genes compared to cluster AM3 (Supplementary Fig. S7; Supplementary Table S5). Cluster AM4 was characterized by higher expression of the glycoprotein Nmb *GPNMB*, galectins; *LGALS3*, *LGALS1* and members of the S100 gene family (*S100A4*, *S100A6* and *S100A11*). Other upregulated genes included superoxide dismutase *SOD2*, phospholipase *PLD3*, cathepsins (*CTSB*, *CTSD*) and the C15orf48 orthologue; *ENSECAG00000012148.* The macrophage scavenger receptor (*MARCO*) transcript levels were notably lower in AM4 compared to all the other macrophage clusters (Fig. 3b). Of note, human monocyte-derived AMs are previously shown to express lower levels of MARCO compared to embryonically derived AMs^35^.

Markers traditionally used to identify pro-inflammatory M1-polarized (*CD68*) and anti-inflammatory M2-polarized macrophages (*CD206*, *CD163*) respectively, were expressed across all the cellular AM subtypes. This observation confirms the emerging view that the classical M1/M2 polarization is an inappropriate classification of AMs. *CD68* expression was moderately higher in the AM4 cluster and concurred with lower *CD206* and *CD163* expression. The reverse was observed in AM3 and AM5 clusters (Fig. 3b). Cluster AM3 exhibited a significant ISG transcript signature (i.e., increased expression of *SAMD9L*, *MX1*, *IFIT2/3*, *XAF1*, *DDX60* etc., Fig. 3b; Supplementary Fig. S7), which is not in accordance with a classical anti-inflammatory M2 phenotype. The most significantly upregulated gene in the AM3 cluster was the membrane skeletal protein *ADD3* (Adductin-3)*.* Other highly expressed genes were the thioredoxin interacting protein *TXNIP*, *RNASE6* as well as complement-system genes Ficolin-1 (*FCN1*|*ENSECAG00000000436*) and *C4BPA.* GO pathway analysis based on the genes upregulated in the AM3 cluster showed enrichment in pathways involved in response to viruses, other organisms, external biotic stimulus and interferon-alpha. In contrast, genes upregulated in AM4 were enriched in pathways related to oxidant detoxification, removal of superoxide radicals and lymphocyte activation (Fig. 3d).

Notably, the cells in cluster denoted AM2 were characterized by an expression pattern comprising elements of both the AM3 and AM4 clusters. Thus, the AM2 cluster cells expressed both higher *GPNMB*, *LGALS4* and *CD68* compared to AM3, as well as higher *MARCO*, *ADD3* and *CD163.* Moreover, expression of ISGs was found within the AM2 cluster (Fig. 3d). The cluster denoted AM5 were mainly characterized by (compared to AM3 and AM4) lower levels of *GPNMB*, *LGALS3*, *ADD3* and ISG transcripts as well as higher expression of e.g., *MRC1* (*CD206*), *CD163*, *ITGAX* (*CD11c*), *DDX21*, *ALOX5AP* and *ENSECAG00000039383* (novel gene, *Wfdc21* orthologue) (Fig. 3b; Supplementary Table S5). The remaining AM clusters (AM1, AM6 and AM7) were rather characterized by overall lower number of genes detected (Supplementary Fig. S7). The small cluster of AM8 were characterized by higher expression of ribosomal protein mRNAs compared to the other AMs.

### BALF mast cell and neutrophil populations

Next, the BALF mast cell and neutrophil populations were examined in more detail. Although the granulocyte recovery was lower compared to the numbers expected from BALF cytology analysis, the single cell transcriptomes of ~ 1250 mast cells (831 cells from mEA, 426 cells from controls) were analyzed. In addition to the markers used for cluster identification shown in Fig. 1, the mast cells in equine BALF expressed high levels of mRNAs in histamine, prostaglandin and leukotriene production pathways (*HDC*, *HPGDS*, *PTGS1* and *LTC4S*). Furthermore, expression of mast cell specific genes, such as *PLCB4*, *RGS13* and *SIGLEC6* was detected (Fig. 1; Supplementary Table S2). Whereas the tryptase *TPSB2* was highly expressed in the mast cells, no expression of chymase (*CMA*) or carboxypeptidase A3 (*CPA3*) was detected. Taken together, this reveals that the mast cells in the lower airways of the equine lung primarily are of the classical mucosal subtype^36^.

**Figure 4:**
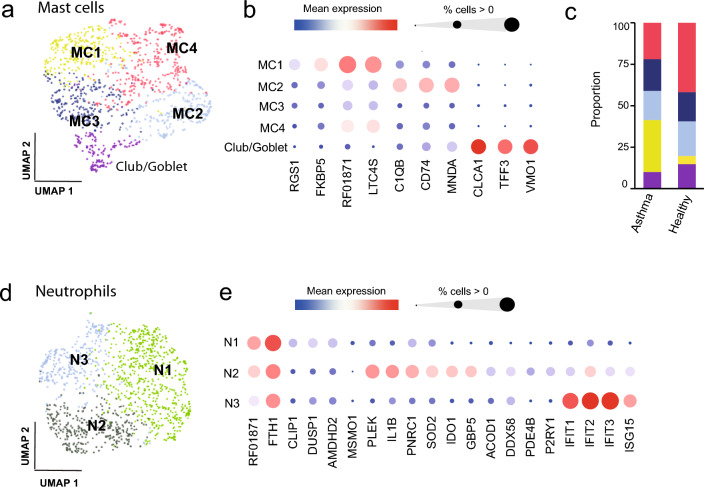
Re-clustering of mast cells and neutrophils. (**a**) UMAP illustration of four mast cell clusters and one cluster comprising club or goblet cells. (**b**) Bubble plot visualizing the expression levels of the top differentially expressed cluster marker genes. The cluster MC1 displays significantly higher expression of *FKBP5*, *LTC4S* and *RGS1.* (**c**) Proportions of mast cell subpopulations in EA and healthy horses: The cluster MC1 (yellow) comprises a significantly higher proportion of cells in horses with asthma (p = 0.001, Kruskal–Wallis). (**d**) Re-clustering of neutrophils results in three subpopulations as illustrated by the UMAP plot. (**e**) Bubble plot visualizing the expression levels of top differentially expressed neutrophil cluster markers and the *FTH1* gene.

Re-clustering of the mast cells resulted in four clusters (Fig. 4a). The clusters were rather similar, although the cells in clusters MC1 and MC2 had the most prominent expression profiles (Fig. 4b; Supplementary Table S6). Cluster MC1 nearly exclusively comprised cells from the EA group (p = 2.2 × 10^−3^, Supplementary Table S3; Fig. 4c; Fig. S8). This cluster was characterized by higher expression of leukotriene C4 synthase (*LTC4S*) as well as the glucocorticoid receptor associated peptidyl-prolyl cis–trans isomerase *FKBP5*. Moreover, regulator of G-protein signaling 1 (*RGS1*), a gene which is associated with auto-immune disorders and potentially also asthma in humans^37,38^, was higher expressed in cluster MC1. In the MC2 cluster we observed complement C1q transcripts (*C1QB* and *C1QC*), *CD74*, *IFI27*, *MNDA* and other genes which are also expressed in macrophages (Fig. 4b; Supplementary Table S6).

A fifth small population (< 100 cells), clustered closely together with the mast cells although absent of typical mast cell signatures. A marker signature including *CLCA1*, *VMO1*, *TFF3*, *SCGB1A1* and *CXCL17* suggests that these cells are club or goblet cells derived from the airway epithelium (Fig. 4b; Supplementary Table S6).

In the initial clustering of BALF cells (Fig. 1), a threshold of > 200 genes/cell was used, resulting in the recovery of approximately 800 neutrophils. Among these neutrophils, several genes such as *CXCL2*, *SNX10*, *CD85* (*ILT11B*), *TREM1*, *CCRL2*, *RGS2*, and *LMNB1* exhibited significantly differential expression compared to other cell types in BALF (Supplementary Table S2 and S6). However, the strict filtering applied during initial clustering may have excluded several neutrophils, owing to their inherently low RNA content. To address this, the BALF cells were re-clustered with a lower threshold of 60 genes/cell (while still being identified as cells by EmptyDrops). This approach yielded around 1270 neutrophils. Within these neutrophils, a generally low number of expressed genes were observed (60–600 genes/cell), which is typical of terminally differentiated granulocyte populations.

Upon re-clustering of the 1270 neutrophils, three different subpopulations were identified (Fig. 4d). The expression of ferritin heavy chain (*FTH1*) was high throughout the neutrophil population and highest in cluster N1 (Fig. 4e), leading us to annotate cluster N1 as FTH1^Hi^ neutrophils^39^. The neutrophil cluster N2 exhibited strong upregulation of ISG expression, while cluster N3 exhibited elevated transcript levels for the pro-inflammatory cytokine *IL-1B*, together with the tryptophan-catabolizing and immunosuppressive *IDO1* and pleckstrin (*PLEK*). Moreover, the activation marker *GBP5*^40,41^, as well as *SOD2* (superoxide dismutase 2), was upregulated in cluster N3, suggesting a pro-inflammatory phenotype (Fig. 4e; Supplementary Table S6).

In summary, despite the rather low number of cells recovered, we identified several distinct subpopulations of mast cells and neutrophils. A subpopulation of mast cells that exhibited higher expression of specific asthma-associated genes was identified, primarily originating from the asthma group. However, there were no discernible differences in the proportion of neutrophil clusters between the asthma and healthy groups (Supplementary Fig. S8; Supplementary Table S3).

### Dendritic cells in equine BALF

**Figure 5:**
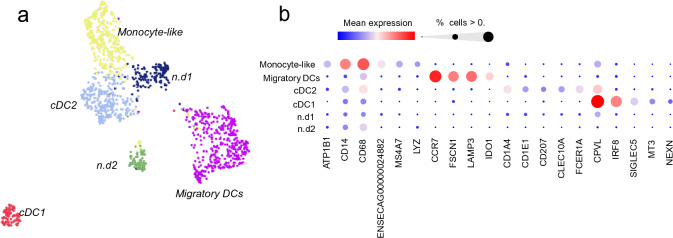
Re-clustering of dendritic cells. (**a**) UMAP illustration of the dendritic cell clusters annotated as conventional DC1 and DC2s, migratory DCs and monocyte derived DCs. (**b**) Bubble plot visualizing the expression levels of the cluster marker genes. *ENSECAG00000024882* = unannotated *CCL15* orthologue. DC = dendritic cells, n.d. = not annotated.

The initial clustering of BALF cells singled out a small population of cells expressing dendritic cell markers (n = 1339 cells, yellow cluster in Fig. 1b, 741 cells from mEA, 598 cells from controls). As dendritic cell populations in the equine lung have not previously been characterized and little is known regarding the subtypes of dendritic cells populating the equine alveolar compartment, we set out to characterize subpopulations based on transcriptome profiles. Independent reiterative clustering of the dendritic cells revealed five putative subpopulations (Fig. 5; Supplementary Table S6). Type-2 conventional dendritic cells (cDC2s) were marked by expression of *FCER1A*, *CD1A*, *CD1E*, *CLEC10A* and *CD207*. Furthermore, we designated a cluster of cells expressing high *ENSECAG00000024882* (novel gene, C–C rich chemokine ligand orthologue), *CD14*, *MS4A7* and *CD68* as monocyte-like cells (putative monocyte-derived DCs)^42^. One cluster with distinct expression of *CCR7*, *FSCN1*, *LAMP3* and *IDO1* was labeled as activated or migratory dendritic cells^42^. A small but distinct cluster of type-1 conventional dendritic cells (cDC1s) exhibited DE genes such as *LRRK2*, *MT3*, *CPVL* and *NEXN.* Two small clusters exhibited weak differential expression of T-cell markers *CD3*, *CXCR6*, *RORA*, *GATA3* (n.d1) and ribosomal protein genes (n.d2), respectively. No labels could confidently be assigned to those two clusters.

### Differential expression analysis reveals upregulation of *FKBP5* and *CCL24* in mEA

Next, DE between EA and healthy controls across different cell types and subpopulations was analyzed. DE analysis was performed at the cell-type level (AMs, T cells and mast cells) using two approaches: (i) a pseudo bulk approach with DESeq2 and (ii) a two-part Hurdle model equivalent to MAST^43,44^. Additionally, DE tests were conducted using two subsets of EA horses: (i) all horses meeting inclusion criteria for mEA compared to controls and (ii) mastocytic mEA cases (Horses N, FS, C, VE, VA, T, D) compared to controls. The results were mostly consistent regardless of which group was compared to the healthy controls. A subset of DE genes is listed in Table S7 and the complete sets of DE genes can be found in Supplementary Tables S8 (AMs), S9 (T cells) and S10 (mast cells and neutrophils).

**Figure 6:**
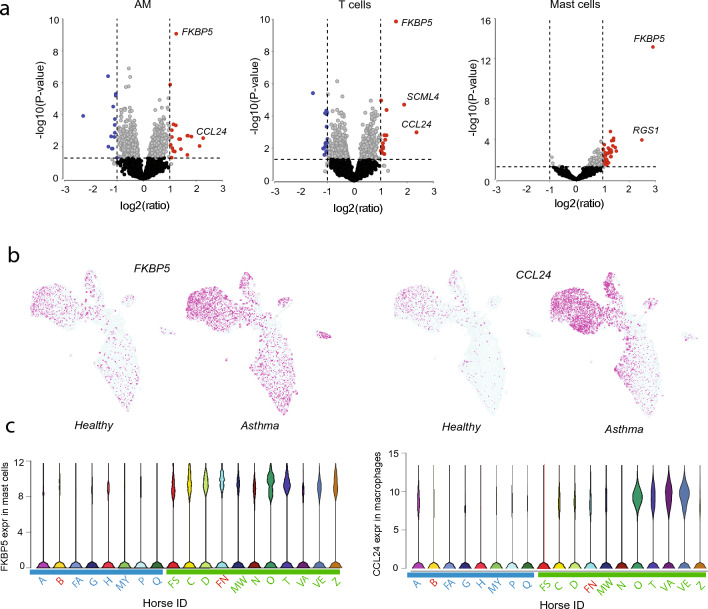
Differential gene expression EA and control horses. (**a**) Volcano plots showing DE genes from pseudo-bulk (DESeq2) analysis of alveolar macrophages (AMs), T cells, and mast cells. The plots show the results from testing the mastocytic group of horses vs controls. (**b**) *FKBP5* and *CCL2*4 expression levels in EA and control cells visualized on the UMAP plot. (**c**) Violin plots showing expression levels of *FKBP5* in mast cells and *CCL24* in alveolar macrophages for individual horses. IDs of healthy horses are denoted in blue and asthma horses denoted in green. (IDs of the horses that did not meet the inclusion critera are marked in red).

Interestingly, all approaches identified *FKBP5* as one of the most significant differentially expressed genes. The highest fold changes were detected in mast cells (DESeq2, all EA cases; log2ratio = 3.0, FDR < 0.001, mastocytic; log2ratio = 2.9, FDR < 0.001) (Fig. 6) while expression in AM and T cells were moderately upregulated (DESeq2 all EA; log2ratio = 1.4 and 1.7, FDR ≤ 0.001 for AMs and T cells, respectively, mastocytic; log2ratio = 1.2 and 1.6, FDR ≤ 0.001). The eosinophil chemotactic protein 2 (eotaxin-2, *CCL24*), was also among the most significant DE genes and highly expressed in AMs derived from EA horses (DESeq2, all EA; log2ratio = 2.1, FDR = 0.1, mastocytic; log2ratio = 2.3, FDR = 0.1). Whilst *FKBP5* upregulation was observed in the majority of mEA horses, *CCL24* expression was primarily upregulated in cells derived from four horses (IDs: O, VA, VE, and T), and the highest upregulation was observed in the AM cluster denoted AM5 (Supplementary Fig. S9; Supplementary Table S8). Although *CCL24* is known to stimulate eosinophilic chemotaxis by interacting with the C–C Motif Chemokine Receptor 3 (*CCR3*)^45^, we did not observe differential expression of this gene in mEA horses with high numbers of eosinophils in BALF compared to mEA horses with low numbers of eosinophils (Supplementary Table S8). Therefore, the gene expression levels of *CCL24* did not appear to correspond with the eosinophil levels in BALF as measured by cytology (Table 1).

Several other significant DE genes were identified, that have previously been associated with asthma in humans or mice. However, most of these genes were detected as significant by only one or the other of the DE approaches (significance threshold; FDR < 0.1, log2ratio ≥ 1, Supplementary Tables S7–S10). For instance, mast cells from asthma horses had higher expression of regulators of G-protein signaling *RGS1* and *RGS13*. Higher expression of thioredoxin interacting protein (*TXNIP*) was observed in mast cells, neutrophils as well as in AMs. Perilipin2 (*PLIN2*) and phospholipid-transporting ATPase (*ATP10A*) were upregulated in AM clusters AM4 and AM5, respectively (Supplementary Tables S7–S10). When using MAST for DE between neutrophils from mEA cases and controls a number of genes previously associated with human asthma were found to be significantly upregulated in the EA group, e.g. *EVI2B*, *RGS2*, *IL4R* and *TXNIP* (FDR < 0.1), Supplementary Table S10)^46–48^. On the other hand, expression of several ISGs (*ISG15*, *IFI27*, *IFI6*) were significantly higher in neutrophils from healthy horses (Supplementary Table S10).

However, upon conducting DE analysis using the pseudo-bulk (DESeq2) approach, no significant gene hits (FDR < 0.1) were observed within the neutrophil cluster. As a result, these findings could not be validated using an alternative DE testing method.

## Discussion

Mild to moderate EA is a prevalent health and welfare concern in horses, leading to poor performance in sport horses^49^. Despite extensive research efforts^4,50^, the underlying mechanisms and precise cellular phenotypes involved in the disease remain largely unknown. While neutrophilic EA is frequently observed in various regions globally, mastocytic mEA, characterized by reduced performance and heightened respiratory effort during exercise, is a prevalent form of EA diagnosed at the University Animal Hospital, Swedish University of Agricultural Sciences (SLU). This study presents a comprehensive scRNA-seq atlas of immune cell populations in equine BALF derived from nineteen clinically evaluated horses with mEA and healthy controls, encompassing a total of 63,022 cells. The study provides a high-resolution cellular composition map of BALF in both healthy and mEA-affected horses. Moreover, *FKBP5*, a protein associated with the glucocorticoid receptor, was identified as a potential biomarker for mEA. This finding may have implications for understanding the underlying mechanisms of mEA and could aid in predicting treatment response for affected horses.

Based on gene expression signatures we identified and categorized the major cell types present in equine BALF, namely, AMs, T cells, DCs, neutrophils and mast cells. The two most abundant cell populations in equine BALF, constituting 90% of cells in the present dataset, were T cells and AMs. A recent similar study by Sage et al. profiled the transcriptomes of 4600 cryopreserved BALF cells obtained from three mEA horses in remission^18^. This study confirms several of their findings and provides additional observations.

Similar to Sage et al., a diverse set of lung resident T cell subtypes were identified, including CD4^+^ helper cells, CD8^+^ cytotoxic cells, and a significant population of previously poorly characterized lung resident equine γδ-T cells^18,51^. However, this study did not observe any NK, NKT, or B-cell clusters, despite their identification in previous scRNA-seq studies of BAL samples^18,52^. Interestingly, inflammatory subsets of T cells (T-ISG^Hi^) that exhibited strong interferon stimulation signatures were identified in all horses. These T-ISG^Hi^ cells were found among both the CD4^+^ and CD8^+^ tissue resident T cell groups. The strongest overall expressions of ISGs were found in T cells and macrophages in two of the EA horses, possibly implying an increased Th1 response. However, this interpretation should be made with caution as it involves only two horses. Previous studies in humans and mice have found that subsets of both mild and severe asthma have increased ISG expression in airways, which was independent of type 2 inflammation and not correlated with the presence of viral infection^53,54^. Future studies using larger numbers of samples are warranted to determine if the T-ISG^Hi^ cells have any functional implications for mEA, and if symptoms are correlated with ISG expression in horses. It is important to note that no viral tests were performed on the study subjects. However, the asthma horses in the study had no signs of infection and had asthma symptoms for several months or years prior to sampling.

Human AMs have been shown to express high levels of *HLA-DR*, *CD11b* (*ITGAM*), *CD206* (*MRC1*), *CD169* (*SIGLEC1*) and *MARCO*^55^. Similarly, we detected high levels of *HLA-DR*, *CD206* and *MARCO* transcripts in equine AMs, as well as moderate expression of *CD11b* and *CD169*. Flow cytometry experiments with human AMs have shown the scavenger receptor *CD163* characterizes two subsets of AMs, and that *CD163*^Hi^ cells were more abundant than the *CD163*^Lo^ cells in BALF^55,56^. Interestingly, the present study observed a similar pattern also in equine AMs. Our study revealed *CD163* expression to be high in the majority of the equine AM populations while one cluster, denoted AM4, exhibited lower *CD163* expression. Moreover, the AM4 cluster was transcriptionally very different from the other AMs, including lower levels of the scavenger receptor *MARCO*. Recent studies have shown that both human blood monocytes and mouse peritoneal macrophages express low levels of *MARCO*^57,58^. It is conceivable that the higher levels of *MARCO* on tissue-resident AM as compared to newly recruited AMs, represents an adaptation to the continuous exposure of the respiratory system to foreign particles.

A substantial number of proliferating AMs was evident in BALF from all horses, in line with the recognized capacity of embryonically derived AMs for self-proliferation. However, it is firmly established that the AM pool can also be replenished through the transformation of monocyte-derived macrophages. The enlistment of fresh AMs from monocyte precursors is a commonly observed phenomenon during instances such as infection and inflammation^55,59^. The cells in the AM4 cluster expressed high levels of glycoprotein Nmb (*GPNMB*) as well as galectins (*LGALS1*, *LGALS3*) genes, which have been shown to be upregulated during macrophage differentiation and in pro-inflammatory response^60,61^. Thus, we suggest that this AM cluster should consist of monocyte-derived AMs, which demonstrate a more pro-inflammatory profile compared to the initial resident AM population. However, this study did not find significantly higher numbers of newly recruited AMs in horses diagnosed with mEA.

Previous studies have reported significant amounts of functional T cell/monocyte complexes in blood, which were found to mark specific immune perturbations^62^. We found a substantial number of cells with expression of both T cell and AM markers (double positive, DP cells). These DP cells presumably constitute doublets, either technical, due to a probability of two cells entering in the same droplet, or they represent cell–cell complexes. To our knowledge, there are no computational methods available to distinguish technical doublets from functional cell–cell complexes. As the number of cells clustering as DP in our analysis were rather high, we are tempted to speculate that a fraction of the DP cells constitutes functional T cell/AM complexes. Immune cells crosstalk is an essential feature of their function, being either performed by signaling molecules or by cells in functional complexes with each other^63^. Nevertheless, macrophages are highly adherent cells and, in future scRNA-seq studies involving equine BALF samples, it may be worthwhile to consider additional strategies in order to ensure dissociation of cell–cell complexes prior to droplet encapsulation. Although the cell composition in the Sage et al.^18^ study data was strongly biased towards lymphocytes and only a few hundred macrophages were analyzed, several observations were still comparable with the present study. While detection of cluster-specific markers was not identical across the studies, both studies identified proliferative AMs, FCN1^High^ AMs (similar profile to the AM3 cluster in the present study), monocyte-derived macrophages, and double-positive cells.

Additionally, we analyzed dendritic and granulocyte cells in BALF. Dendritic cell populations in the equine lung have not previously been characterized and little is known regarding the subtypes of dendritic cells populating the equine alveolar compartment. Several functional classes of dendritic cells are found in humans, and some of those have previously been investigated in single-cell studies of BALF and other lung samples^42,64^. We identified conventional DCs as well as migratory and monocyte-derived DCs in equine BAL. In support of our annotations of the equine lung dendritic cell populations, we found that the top markers identified for the different subclasses of dendritic cells in our study agree well with those identified for the human equivalents in single cell studies of the lung^42^.

Mast cells in equine BALF were found to be exclusively of the mucosal type^65^, although several sub-clusters were found. Interestingly, a small subpopulation of mast cells comprised mostly of cells from asthma horses and displayed higher expression of e.g., the asthma associated genes *FKBP5*, *RGS1* and *LTC4S*, suggesting a role in inflammation.

Neutrophils, which are terminally differentiated cells, have traditionally been considered to be rather homogenous, but recent studies have shown that they can have distinct subpopulations^66^. For instance, low-density neutrophils (LDNs) are known to be a subset of neutrophils associated with several human inflammatory conditions and a recent study also found increased levels of LDNs in the blood of horses with sEA^67^. In the present study, we found several distinct subpopulations of neutrophils, including those with upregulated ISGs and those with differentially expressing genes related to inflammation and activation^40,41^. However, we did not observe any significant differences in neutrophil composition or gene expression between cases and controls, which may be due to the small number of cells, and the fact that the cases enrolled in the study were not typical neutrophil horses. The ferritin heavy chain gene (*FTH1*) was highly expressed throughout the whole neutrophil population. *FTH1* is a cytosolic iron storage protein that is believed to also be involved in physiological processes related to oxidative stress and inflammation. A recent study in mice lungs found that *FTH1* was highly expressed in neutrophils within the bronchi and alveolar space but not in neutrophils from pulmonary blood vessels, and that the *FTH*^Hi^ phenotype in mice displayed increased oxidative resistance, delayed apoptosis, and greater production of inflammatory mediators^39^. Although not directly applicable to the current context, it's noteworthy that a single-cell RNA study focusing on neutrophils in the peripheral blood of human sepsis patients unveiled various distinct subgroups of neutrophils. Two of the subgroups exhibited similarities with neutrophil clusters identified in this study: one was distinguished by the expression of *FTH1*, and another displayed an increase in ISGs^68^. Given that rather small populations of these cell types were recapitulated and analyzed herein, further studies will be necessary to investigate the functional implications and impact of the identified subpopulations and differentially expressed genes on equine airway disease.

We identified highly significantly DE gene transcripts in clinical cases of mEA compared to healthy controls, which potentially can be further explored as potential biomarker for asthma and therapy response in horse and man. *FKBP5* was one of the most significant DE genes in all cell types analyzed (AMs, T cells and mast cells), with the highest upregulation observed in the mast cells (Supplementary Table S7). Although several studies have examined gene expression differences between healthy and asthmatic horses, to our knowledge, there are no previous reports demonstrating any associations between *FKBP5* and EA^15,69^. The FKBP5 protein acts as a co-chaperone and modulates the glucocorticoid receptor activity by binding to other co-chaperones, e.g., the heat shock protein 90 (Hsp90) and P23 protein, which are important for the function of the glucocorticoid receptor^70,71^. Hsp90 is one of the two main chaperone machines that influences glucocorticoid receptor assembly and activity, together with Hsp70^72^. By interacting with this complex, FKBP5 can modulate sensitivity of the glucocorticoid receptor. It has been demonstrated that increasing levels of FKBP5 reduces the transcriptional activity of the glucocorticoid receptor and that the binding of FKBP5 to Hsp90 affects cortisol affinity to the receptor^73^. In squirrel monkeys, it has been reported that corticosteroid resistance is linked to overexpression of FKBP5^74–76^. In humans, about 10–20% of patients with inflammatory diseases and immune disorders do not respond to treatment with glucocorticoids^77^. In addition, corticosteroid insensitivity (i.e., an impaired response to corticosteroids) in human asthma is a well-recognized phenomenon, and it has been estimated that corticosteroid insensitivity is present in approximately one-third of asthma patients^78^. Although equine blood neutrophils have been found to be sensitive to glucocorticoids^79^, we are not aware of any other published reports regarding the prevalence of corticosteroid resistance or insensitivity in horses. Mastocytic mEA is associated with airway hyperreactivity, and affected horses often demonstrate limited response to corticosteroid treatment. For those horses, mast cell stabilizing drugs are sometimes suggested as an alternative treatment option. Considering this, the increased expression of *FKBP5* transcripts in lung immune cells of mEA horses with a mast cell phenotype is an interesting finding, which possibly relates to steroid insensitivity in equines. It should, however, be mentioned that several factors, including disease severity, concurrent disease, antigen exposure, as well as the compliance and administration techniques by the caregivers, may have an effect on treatment response^80^. As such, further studies are needed to fully understand the role of FKBP5 in asthma, as well as in corticosteroid response in horses.

A second significantly DE gene, particularly highly expressed in AMs from mEAS horses, was *CCL24*, also known as eotaxin-2. *CCL24* is a cytokine belonging to the C–C chemokine family. It interacts with the C–C Motif Chemokine Receptor 3 (*CCR3*) and stimulates eosinophilic chemotaxis^45^. Several studies have demonstrated significant associations between the *CCL24* gene and allergic human asthma, using airway epithelial cells and sputum^45,81–84^. However, in BALF samples, while the levels of eotaxin-2 mRNA were higher than in the airway epithelial cells, the levels did not differ between asthmatic patients and healthy controls^45^. The protein levels for eotaxin-2 were actually lower in the severe asthma cases, compared the healthy controls, and the severe group did not differ from the milder asthma groups^45^. In horses, two studies have suggested a possible impact of *CCL24* on sEA^14,85^. While the *CCL24* gene may be important for lung eosinophilia^86^, the current study did not observe any differences in expression of *CCL24* when comparing asthmatic horses with high numbers of eosinophils to asthmatic horses with low numbers of eosinophils. Given that *CCL24* expression was highly upregulated in cells derived from a subset of the mEA horses, it will be interesting to further investigate expression of this gene and how it varies in different EA phenotypes.

Several limitations can be identified in our study. ScRNA-seq experiments performed on non-model organisms faces several challenges. One of the hurdles is that the equine reference genome is currently not as well annotated as the human or mouse genomes. Moreover, cell type annotation in horses is a laborious manual process, as reference sets of cell-type markers are not yet available for this species, and especially T cell subpopulations are challenging to annotate manually^18–20^. Another limitation of this study is the rather low sensitivity of the Drop-Seq technology, which restricts the number of genes that can be detected per cell. Droplet-based technologies are also not optimal for analyzing sensitive cells such as granulocytes due to (i) the fragility of these cells which may not survive the encapsulation process (ii) their low expression levels leads to losses during data pre-processing and quality filtering. As a result, granulocytes are often underrepresented in droplet-based scRNA-seq data^87^. However, as the field evolves toward more sensitive methods and alternative cell isolation technologies adapted for fragile cells (e.g., where cells settle gently into picowells by gravity), we can expect an increased resolution of cell-types and cell states in future scRNA-seq studies of EA. The cases included in the study were clinical patients with a history and anamnesis of mEA. Blood analysis (e.g., inflammatory markers) and thoracic radiography were not conducted for all cases to exclude potential differential diagnoses, such as sEA or equine multinodular pulmonary fibrosis. Lastly, due to the high costs of the technology, the number of horses included in the study is limited.

## Conclusion

In summary, this study is the first to provide an unbiased characterization of immune cells present in the horse airway under both healthy and diseased conditions using scRNA-seq. The detailed description of cell types in BALF will serve as a valuable resource for future studies of EA. The similarities between our findings and the immune cell subsets found in human BALF suggest that mEA may be a useful model for the human condition. Importantly, this study detects the glucocorticoid receptor-associated protein *FKBP5* and *CCL24* as potential biomarkers for EA. Further investigations will be needed to determine if *FKBP5* and *CCL24* are associated with disease severity and if *FKBP5* correlates with treatment response.

## Methods

### Animals

All aspects of the study that involve sampling of horses have been approved by the Swedish ethical review board (# 5.8.18-20690/2020). All horse owners approved the study by signing a written consent form. All methods were carried out in accordance with relevant Swedish guidelines and the ARRIVE guidelines were followed. In total nineteen horses (eleven client-owned clinical cases, eight control horses) were included in the study. The asthma horses were clinical patients presented to and examined at the University Animal Hospital, SLU in Uppsala. The control horses were experimental horses owned by Department of Clinical Sciences, Swedish University of Agricultural Sciences. The included control horses were sampled in April had no history of clinical symptoms of EA and were not treated before the experiment. The inclusion criteria for the horses with mEA were according to consensus: (i) elevated granulocytes in BALF (> 10% neutrophils and/or ≥ 5% eosinophils, and/or ≥ 4% mast cells) (ii) chronic symptoms of mEA (e.g. intermittent coughing and/or exercise intolerance ≥ 4 months) (iii) no signs of infection (such as fever and lethargy). The inclusion criteria for control horses were: (i) normal BALF cytology (ii) no symptoms or clinical history of EA or other disease, (iii) no signs of infection, and normal endoscopic findings. A comprehensive description of both cases and controls, encompassing clinical scoring, clinical history, type of mEA, ongoing medications, age, and breed, can be found in Supplementary Information S1 (Study Design), Supplementary Fig. S1 and Supplementary Table S1. One horse with normal BALF cytology (Horse FN) in the asthma group and one control horse with elevated BALF neutrophils (Horse B) did not meet the inclusion criteria and were therefore excluded from all differential gene expression tests between cases and controls (but included in the cell type characterization).

### Method details

#### Endoscopy examination and BALF sampling

All horses received detailed clinical respiratory examinations and airway endoscopy. The healthy control horses showed no signs of airway inflammation, as judged by endoscopy examination. Before collection of BALF, the horses received intravenous premedication with sedative (detomidine and butorphanol tartrate) and a local anaesthetic (Carbocain®) was instilled at the trachea. The BALF sampling was performed with a blind tube with 3 × 100 ml sterile isotonic saline solution (at 37 °C).

#### Cytology analysis

A 15 ml aliquot of BALF was transferred to the Clinical Pathology Laboratory, University Animal Hospital, SLU, and processed within four hours. The total number of nucleated cells was determined using Advia 2120 (Siemens Healthcare GmbH, Ashburn, Germany). For cytological evaluation of BALF samples, cytospin preparations were prepared using two different concentrations of BALF. The first was prepared by centrifuging a 10 ml aliquot at 500×*g* for five mins and resuspending the resulting cell pellet with 50 μl albumin solution (1 g bovine serum albumin and 0.002 g NaN_3_ dissolved in 10 ml of 0.9% NaCl). The uncentrifuged preparation was made by adding 50 μl of the albumin solution to a 200 μl aliquot of the original BALF. Cytospin preparations with 100 μl aliquots were prepared in cytocentrifuge cassettes (Thermo Scientific Cytospin 4 centrifuge, Thermo Fisher Scientific, Waltham, Massachusetts, US). The slides were stained with May-GrÜnewald-Giemsa and evaluated by a clinical pathologist. A 200 differential count was performed, where cells were classified as macrophages, lymphocytes, neutrophils, mast cells or eosinophils, expressed as a percentage. Epithelial cells, which often appear in aggregates, and non-intact cells were excluded from the differential count.

#### Preparation and sequencing of Drop-Seq libraries

Cells were kept at 4 °C and on ice during transport. The samples were processed as soon as possible, but no later than four hours, after the BALF sampling. Single cells were encapsulated together with microbeads (ChemGenes Corporation, Wilmington, MA, USA) in droplets on the Nadia microfluidic device (Dolomite Bio, Royston, UK), according to the Drop-Seq protocol^21^, with some minor modifications. Briefly, microbeads were resuspended in cell-lysis buffer prior to droplet encapsulation. Cell suspension concentrations were kept low (300 cells/ul), to avoid macrophage aggregation. The cell suspension was counted with the Cellometer K2 (Nexcelom Bioscience, Lawrence, MA, USA) using AO/PI staining solution. The cell viability was > 90% in all samples. The microfluidic cartridge was loaded with 250 µl of cell suspension (300 cells/ul) and 250 µl of bead suspension (600 beads/ul). Magnetic stirrers were used in the sample compartment of the Nadia Dolomite microfluidic cartridge. After droplet encapsulation, the resulting emulsion was collected and broken by filtration through a 5 μm Uberstrainer filter (PluriSelect, Leipzig, Germany). The suspension was added to the filter and 45 ml of 6xSSC buffer was passed through the filter, with the help of a 50 ml syringe. The beads were then collected from the filter, resuspended in high-salt 6xSSC buffer, spun down and washed with 5× Maxima RT buffer (Thermo Fisher Scientific, Waltham, MA, USA). The beads were resuspended in 200 µl reverse transcription (RT) reaction mix containing: 1× Maxima H Minus RT buffer, 4% Ficoll PM-400, 2.5 µM TSO oligo, 1 mM dNTP, RNase Inhibitors (RNaseOut and SUPERaseIn) and 2000 U Maxima H Minus Reverse Transcriptase (Thermo Fisher Scientific, Waltham, MA, USA). The RT mix was incubated 30 min at 20 °C followed by 90 min at 42 °C and gentle shaking to keep the beads in suspension. After RT, Exonuclease I was added, to digest excess oligos, and incubated at 37 °C for 30 min. The beads were spun down (1000 g, 1 min) and washed, first in 1 ml TE-SDS buffer, then in 1 × 2 ml TE-Tween buffer and finally resuspended in H_2_0. Beads were counted, aliquoted in a 96-well plate (5000 beads/well) and 14 cycles of cDNA amplification was then carried out using Terra PCR polymerase (TakaraBio, Kusatsu, Japan) and SMART PCR oligo. Amplified cDNA was pooled and purified twice with 0.6× AMPure beads (BeckmanCoulter, Brea, CA, USA) and the size profiles were determined on Tape station (size range 400–3000 bp). The concentration of amplified cDNA was measured with Qubit and 1 ng was used as input for Nextera XT library preparation (Illumina, San Diego, CA, USA) using New P5 SMART PCR oligo and Nextera index oligos. Phusion polymerase (Thermo Fisher Scientific, Waltham, MA, USA) was used for library amplification (10 PCR cycles). The number of beads corresponding to ~ 6000 cells/sample were taken into the amplification and library preparation steps. Three Nextera libraries per sample were prepared and pooled. Sequencing libraries were quantified using qPCR and sequenced on the NovaSeq6000 instrument (Illumina, San Diego, CA, USA) using custom sequencing primer (Read1CustomSeqB) and aiming at a read depth corresponding to > 50,000 reads/cell (Supplementary Table S11). Primer sequences are listed in Supplementary Table S2.

#### Primary data analysis and filtering

Data analysis and visualizations were performed using the Partek Flow single cell analysis framework (Partek Inc, Chesterfield, MO, USA). The Drop-Seq toolkit^21^ implemented in Partek Flow were used to (i) trim reads, (ii) perform alignment to the EquCab3.0 (Ensembl release 97) using STAR v2.5.3a, (iii) deduplicate UMIs, (iv) filter and quantify barcodes to transcriptome. The EmptyDrops^88^ test was used to distinguish real cells from empty droplets, resulting in a recovery of between 2538 and 5757 cells per horse, in total 72,000 cells. Cells with fewer than 200 detected genes or with > 5% mitochondrial reads were excluded. Potential doublets were identified and removed based on UMI counts > 8000. This resulted in removal of 4% of the sequenced cells (~ 3000 cells). After stringent quality filtering, 63,000 cells remained for downstream analysis and clustering, in which between 200 and 1620 genes/cells were detected. Genes with zero expression in > 99.9% of cells were filtered out, to reduce noise.

#### Cluster analysis

Normalization (log2(CPM + 1)) and scaling was performed, followed by dimensional reduction in PCA. The number of principal components used in downstream clustering was determined based on Scree plots. Harmony was used for batch correction and integration of data from all horses^89^. Graph-based clustering and UMAP were performed to visualize and annotate cell types^22,90^. When re-clustering cell types, which comprised only a small number of total cells, the Harmony integration resulted in large numbers of non-informative clusters. The Harmony integration step was therefore omitted when re-clustering mast cells, neutrophils and DCs. Cluster biomarkers were computed by comparing the gene expression in every cluster to the other clusters combined, using the ‘compute biomarker’ function (t-test) implemented in Partek Flow. Differential gene expression between clusters or groups of clusters was also analyzed using the two-step hurdle model implemented in Partek Flow, which is equivalent to the MAST (‘Model-based Analysis of Single-cell Transcriptomics’) method^43^. Cell types were manually annotated based on the expression of previously known and canonical cell type markers from studies of immune cells in humans and other animal models. Additional information regarding analysis parameter settings is listed in Supplementary Table S12. The assumption of normality was evaluated using the Shapiro–Wilk test. As cell proportions did not exhibit a normal distribution in both the case and control groups a non-parametric test (Kruskal–Wallis) was performed in order to test if cell group sizes (in %) were significantly different in EA vs healthy controls (Table 1 and Supplementary Table S3). Heat maps and bubble plots were made in Partek Flow and the heat maps clustered by horse ID within each cell type or subpopulation (in order; A, FS, B, C, D, FA, FN, G, H, MW, MY, N, O, P, Q, T, VA, VE, Z).

#### Differential gene expression between conditions

DE analysis across the EA and healthy groups was performed using (i) a pseudo bulk approach and (ii) a method specifically adapted for analyzing single cell data (two-step hurdle model, MAST)^43^ as implemented in the Partek Flow software framework. For the pseudo bulk analysis, the gene expression values were pooled for every cell type or cluster analyzed, and differential gene expression across groups were computed using DESeq2. P-values were corrected using FDR multiple test correction^91^. Additional information regarding parameter settings is found in Supplementary Table S12. GO enrichment analysis was performed with g:Profiler^92^.

### Supplementary Information

Supplementary Information 1.

Supplementary Table S2.

Supplementary Table S3.

Supplementary Table S4.

Supplementary Table S5.

Supplementary Table S6.

Supplementary Table S8.

Supplementary Table S9.

Supplementary Table S10.

Supplementary Table S11.

Supplementary Table S12.

## Data Availability

The scRNA-seq data have been deposited at SRA, accession #PRJNA914226 and is publicly available.
