# Supplementary material for: Single-cell transcriptomics delineates the immune cell landscape in equine lower airways and reveals upregulation of FKBP5 in horses with asthma
Source: Sci Rep. 2023 Sep 27;13:16261. doi: 10.1038/s41598-023-43368-4 (PMC10533524; doi:10.1038/s41598-023-43368-4)
Supplement: Supplementary file 1 — Supplementary Information 1. [file 41598_2023_43368_MOESM1_ESM.pdf]

# Single-cell transcriptomics delineates the immune cell landscape in equine lower airways and reveals upregulation of FKBP5 in horses with asthma

Miia Riihimäki[1], Kim Fegraeus[2], Jessica Nordlund[2], Ida Waern[3], Sara Wernersson[3], Srinivas Akula[3], Lars Hellman[4] and Amanda Raine[2]*

## Supplementary Information

Supplementary S1 Study Design
Supplementary Table S1 (Clinical description), Table S7 (Examples of DE genes)
Supplementary Figure S1-S9
Supplementary Table S2 Cell type cluster markers .
Supplementary Table S3 Compositional analysis statistics.
Supplementary Table S4 T cell type cluster markers.
Supplementary Table S5 Alveolar macrophage cluster markers.
Supplementary Table S6 Mast cell, neutrophil & dendritic cell cluster markers.
Supplementary Table S7 (Examples of DE genes)
Supplementary Table S8 Lists of DE genes, AMs asthma vs healthy.
Supplementary Table S9 Lists of DE genes, T cells asthma vs healthy.
Supplementary Table S10 Lists of DE genes, mast cells & neutrophils asthma vs healthy.
Supplementary Table S11 Primer sequences
Supplementary Table S12 Analysis parameter settings.

## Study design

*Clinical and endoscopic scoring*

All horses were examined and scored by the same veterinarian. The clinical scoring system used was the same as in[1,2], but tracheal auscultation was replaced with pulmonary auscultation with a rebreathing bag, scoring 0 as normal and 1 when crackles or wheezes were present. Maximal total score was 20. Upper and lower airway endoscopy was performed after sedation of the horses. The pharynx, guttural and trachea were examined endoscopically and the degree of pharyngeal lymphoid hyperplasia was scored[3]. Mucus in trachea was quantified using a previously published 5-grade system[4].

*Control horses*

The eight control horses included in the study were part of the research herd owned by the Department of Clinical Sciences, Swedish University of Agricultural Sciences (SLU) in Uppsala. All horses were Standarbred trotters, six mares and two geldings, and had a mean age of 13,5 years (SD±3.5) (Table 1). The inclusion criteria required that the horses had no clinical history of symptoms indicating equine asthma (EA), a clinical score of 0[1], no endoscopic findings indicating any disease and no signs of infection (such as fever). One of the control horses (Horse A) had cytological findings in BALF indicating increased neutrophils, but it had no other symptoms. This may have indicated subclinical EA or seasonal variation due to stabling, but Horse A did not meet the inclusion criteria for the control horses and was therefore excluded when analyzing differential gene expression between cases and controls.

All horses were housed in a modern stable in individual boxes bedded with wood shavings and were fed haylage. Water was available ad libitum. Clinical examinations, endoscopic examinations of upper airways, guttural pouches, and trachea as well as BAL was performed at the sampling time (in the end of the stabling season Figure S1).

*Equine asthma horses*

Eleven horses with a mean age of 13.3 years (SD±6.3), and with a history and clinical signs indicating EA, were included in the study (Table S1). The horses were sampled on convenience occasions during the years 2020-2021 when cases presented to the clinic and coincided with the availability of laboratory staff to perform scRNA-seq sample preps. All horses were sampled during the stabling period (Figure 1). All horses were client-owned and were sampled and examined at the University Animal Hospital (UDS, SLU, Uppsala) due to their history and clinical signs of EA. The horses (except individual FN) had several months to years of history of coughing without fever and had increased granulocytes in BAL at the sampling occasion (main Table 1). The inclusion criteria were[6]: i) > 10% neutrophils and/or ≥ 5% eosinophils, and/or ≥ 4 % mast cells in BALF, ii) chronic symptoms of mEA (intermittent coughing and/or exercise intolerance ≥ 4 months), iii) no signs of infection (such as fever and lethargy). Horse FN did not have a history of coughing but had a history of several years of seasonal (spring) exercise intolerance, unexpected hyperpnea, and sneezing. Since horse FN had normal BAL cytology at the sampling occasion, this horse did not meet the inclusion criteria and was therefore excluded when analyzing differential gene expression between cases and controls.

The BALF samples were collected after clinical indication of EA and sent for both routine cytological analysis and transcriptomic analysis at the same time. Cells were further prepared for transcriptomic analysis prior to obtaining cytology results in order to maximize the viability of the cells analyzed.

Horse Z had, according to the owner, been treated with inhalation therapy (fluticasone propionate) 32 days prior to examination and sampling. Moreover, two horses were treated with allergen-specific immunotherapy (ASIT) when sampled: FN (with a history of seasonal symptoms) and FS (with a previous history of allergic dermatitis).

Three horses (VA, T, and Z) were kept in outdoor shelters with an open barn system, using straw shavings as bedding. The remaining horses were stabled with straw shavings, except for one horse (FS) that had peat shavings. Six of the horses were fed with hay, and five with haylage (FN, D, T, C, and VE). A summary of the horses included in the study and their diagnostic details are shown in Table S1.

*Additional comments*

To ensure the viability of the cells for successful scRNA-seq analysis, BALF samples were processed for transcriptomic analysis prior to receiving cytology results. Mastocytic inflammation was the most commonly observed finding in EA horses at UDS over the last few years, and further investigations are required to determine why this phenotype is predominant at UDS and generally prevalent in Sweden and other Scandinavian countries. The horses included in the study were a mix of referral and first opinion cases.

Although the horses were not sampled for viral infections in this study, they all had several months of clinical history of coughing and/or exercise intolerance, indicating non-infectious inflammation. Moreover, even healthy asymptomatic horses can test positive for viral infections[5], implying that a positive viral test does not automatically indicate that the detected inflammation in BALF cytology is caused by a current viral infection.

## References

1. Ivester, K. M., Couëtil, L. L. & Moore, G. E. An observational study of environmental exposures, airway cytology, and performance in racing thoroughbreds. *J. Vet. Intern. Med.* **32**, 1754–1762 (2018).

2. Tesarowski, D. B., Viel, L. & McDonell, W. N. Pulmonary function measurements during repeated environmental challenge of horses with recurrent airway obstruction (heaves). *Am. J. Vet. Res.* **57**, 1214–1219 (1996).

3. Raker, C. W. & Boles, C. L. Pharyngeal lymphoid hyperplasia in the horse. *J. Equine Med. Surg.* **2**, 202–207 (1978).

4. Gerber, V. *et al.* Endoscopic scoring of mucus quantity and quality: observer and horse variance and relationship to inflammation, mucus viscoelasticity and volume. *Equine Vet. J.* **36**, 576–582 (2004).
5. Back, H. *et al.* Viral load of equine herpesviruses 2 and 5 in nasal swabs of actively racing Standardbred trotters: Temporal relationship of shedding to clinical findings and poor performance. *Vet. Microbiol.* **179**, 142–148 (2015).
6. Couëtil, L. L. *et al.* Inflammatory Airway Disease of Horses—Revised Consensus Statement. *J. Vet. Intern. Med.* **30**, 503–515 (2016).

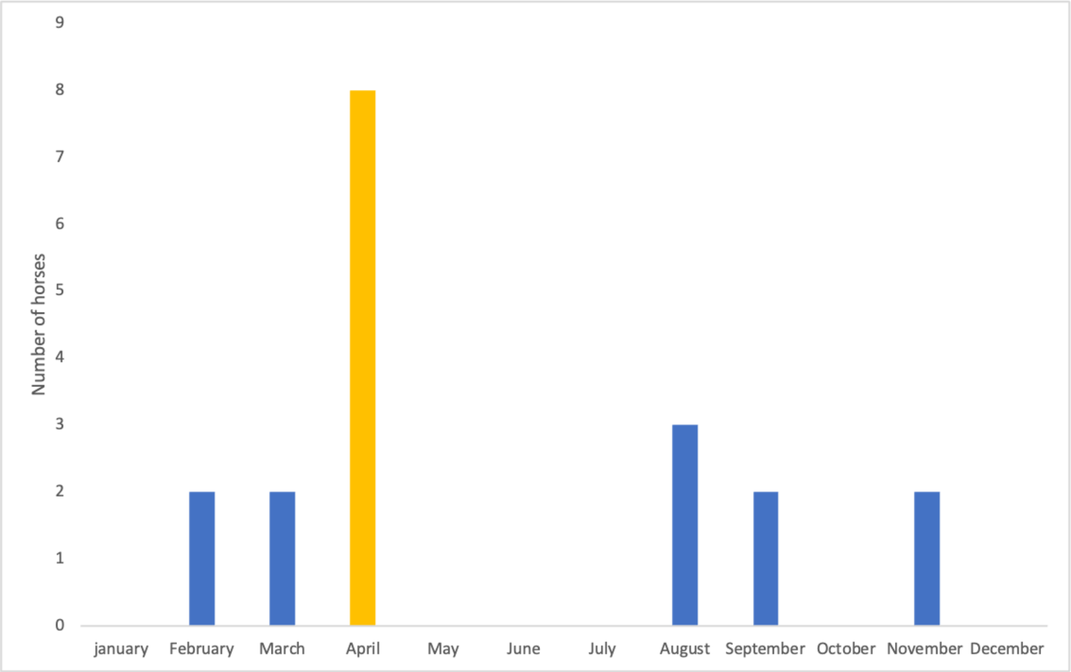


**Figure S1**. All horses with history of equine asthma (blue staples) were sampled during the stable period and the control horses (yellow staple) were sampled in end of the stabling period (April)

**Table S1**

| ID | Phenotype | Age | Sex[7] | Breed[8] | Clinical scoring[9] | Mucus scoring[10] | Type of mild/moderate EA | History | Duration of clinical signs prior sampling |
|---|---|---|---|---|---|---|---|---|---|
| MW | Asthma | 13 | M | CP | 0 | 2 | Mixed neutrophilic | Intermittent coughing, unexpected hyperpnea[11], exercise intolerance | >5 months |
| O | Asthma | 9 | G | CBT | 0 | 1 | Mastocytic and eosinophilic, hemosiderin in macrophages | Coughing during exercise | 5-6 months |
| Z | Asthma | 14 | G | NSH | 0 | 1 | Mixed neutrophilic | Intermittent coughing and nasal discharge | 4 months |
| N | Asthma | 4 | M | SB | 0 | 1, pharyngitis grade 1 | Mastocytic and eosinophilic, hemosiderin in macrophages | Unexpected hyperpnea, earlier coughing | 1 year |
| FS | Asthma | 18 | G | SRP | 0 | 1 | Mastocytic and eosinophilic | Intermittent coughing | 5-6 months |
| C | Asthma | 12 | M | SW | 0 | 2 | Mastocytic | Intermittent coughing, exercise intolerance | 4-5 months |
| VE | Asthma | 25 | G | WM | 0 | 3 | Mastocytic | Intermittent coughing, PPID | 1.5 years |
| VA | Asthma | 6 | M | T | 0 | 1 | Mastocytic and eosinophilic | Intermittent coughing, unexpected hyperpnea, exercise intolerance | 1 year |
| T | Asthma | 21 | M | IH | 2 | 1 | Mastocytic, hemosiderrin in macrophages | Coughing and unexpected hyperpnea | Several years |
| D | Asthma | 15 | G | SW | 1 | 1 | Mastocytic | Coughing during exercise, exercise intolerance | Several years |
| FN | n.d[12] | 9 | G | SW | 1 | 0 | Normal BALF Cytology | Summer seasonal symptoms: unexpected  hyperpnea, sneezing and | Several years |

| | | | | | | | | |
|---|---|---|---|---|---|---|---|---|
| | | | | | | | | exercise intolerance. Sampled due to clinical signs early spring |
| MY | Control | 14 | M | SB | 0 | 0 | Normal BALF Cytology | |
| A | Control | 13 | G | SB | 0 | 1 | Normal BALF Cytology | |
| B | n.d[12] | 10 | M | SB | 0 | 0 | Neutrophilic | Asymptomatic mild EA or seasonal neutrophilic inflammation? No clinical signs |
| F | Control | 12 | M | SB | 0 | 0 | Normal BALF cytology | |
| H | Control | 16 | G | SB | 0 | 0 | Normal BALF cytology | |
| Q | Control | 8 | M | SB | 0 | 0 | Normal BALF cytology | |
| G | Control | 17 | M | SB | 0 | 0 | Normal BALF cytology | |
| P | Control | 18 | M | SB | 0 | 0 | Normal BALF cytology | |

7. M: Mare, G: Gelding
8. CBT: Coldblooded trotter, CP: Connemara pony, IH: Icelandic horse, NSH: North-Swedish draught horse, SB: Standardbred, SRP: Swedish riding pony, SW: Swedish Warmblood, T: Tinker, WM: Welsh mountain
9. Clinical scoring (modified from[1,2])
10. Endoscopic quantitative tracheal mucus scoring[4]
11. Unexpected hyperpnea = exaggerated respiratory effort during work.
12. n.d = phenotype not defined, did not meet inclusion criteria, excluded from DE testing.

**Table S1**. Summary of horses included in the study, definition of asthma based on BALF cytology according to[6]

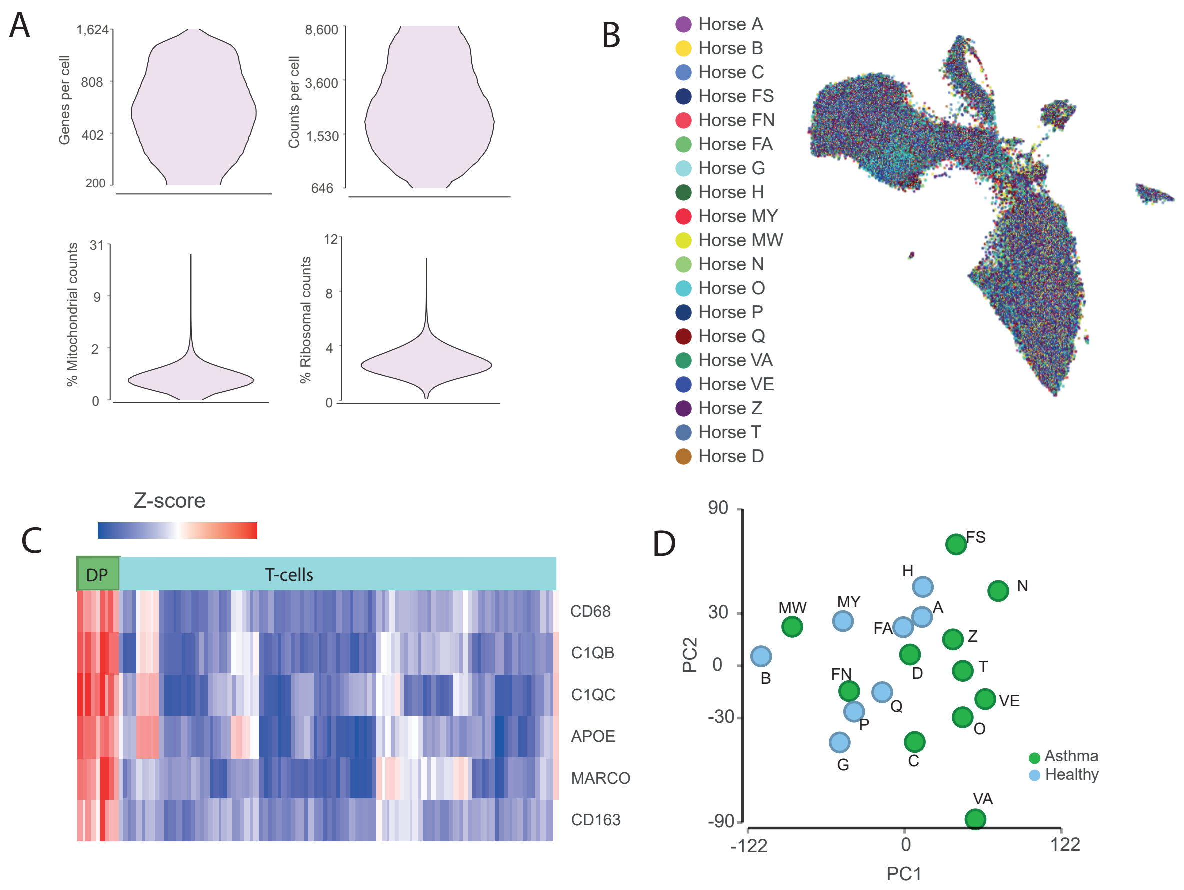


**Figure S2**.
A) Quality control metrics shown for the 63,022 BALF cells after excluding low quality cells. B) UMAP visualization of the integrated clustering of 63,022 BALF cells after batch correction using Harmony. Cells are colored by horse ID.
C) Double positive (DP) cells clustered with the T-cells and simultaneously demonstrated expression of macrophage markers. 2800 DP cells were excluded in downstream analysis. D) PCA plots showing sample-level gene expression variation.

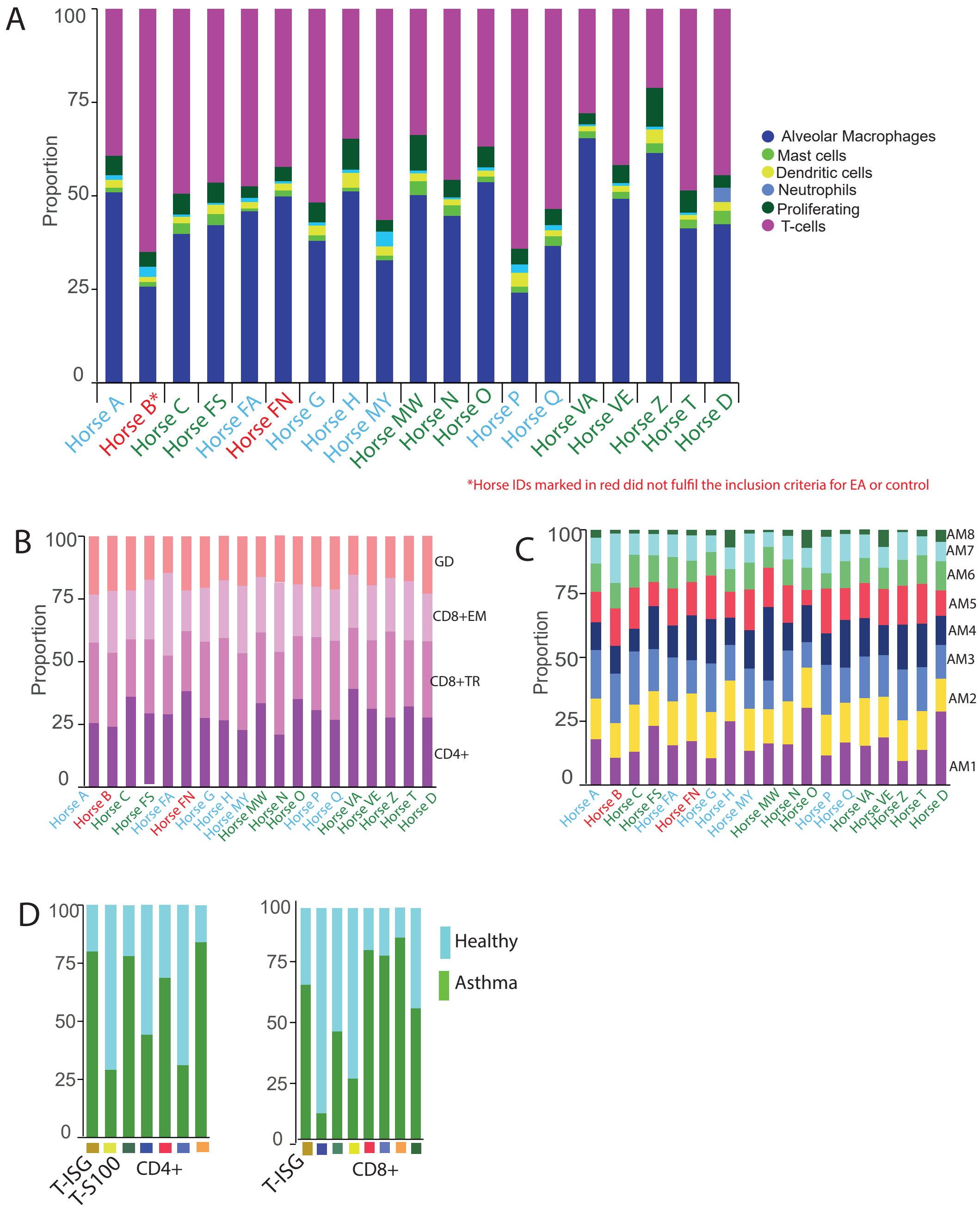


**Figure S3**.
A) Proportions of major cell-types in individual horses (as identified by scRNA-Seq).
B) Proportions of T-cell subtypes in individual horses (GD = gamma delta T cells, EM = effector memory, TR = tissue resident).
C) Proportions of alveolar macrophage clusters in individual horses.
D) Proportion of cells derived from asthma horses (green bars) and healthy horses (light blue bars) in subclusters of $CD4^{+}$ and $CD8^{+}_{TR}$ cells, respectively. Colors below the bars indicate which cluster they correspond to in Figures 2D and E.

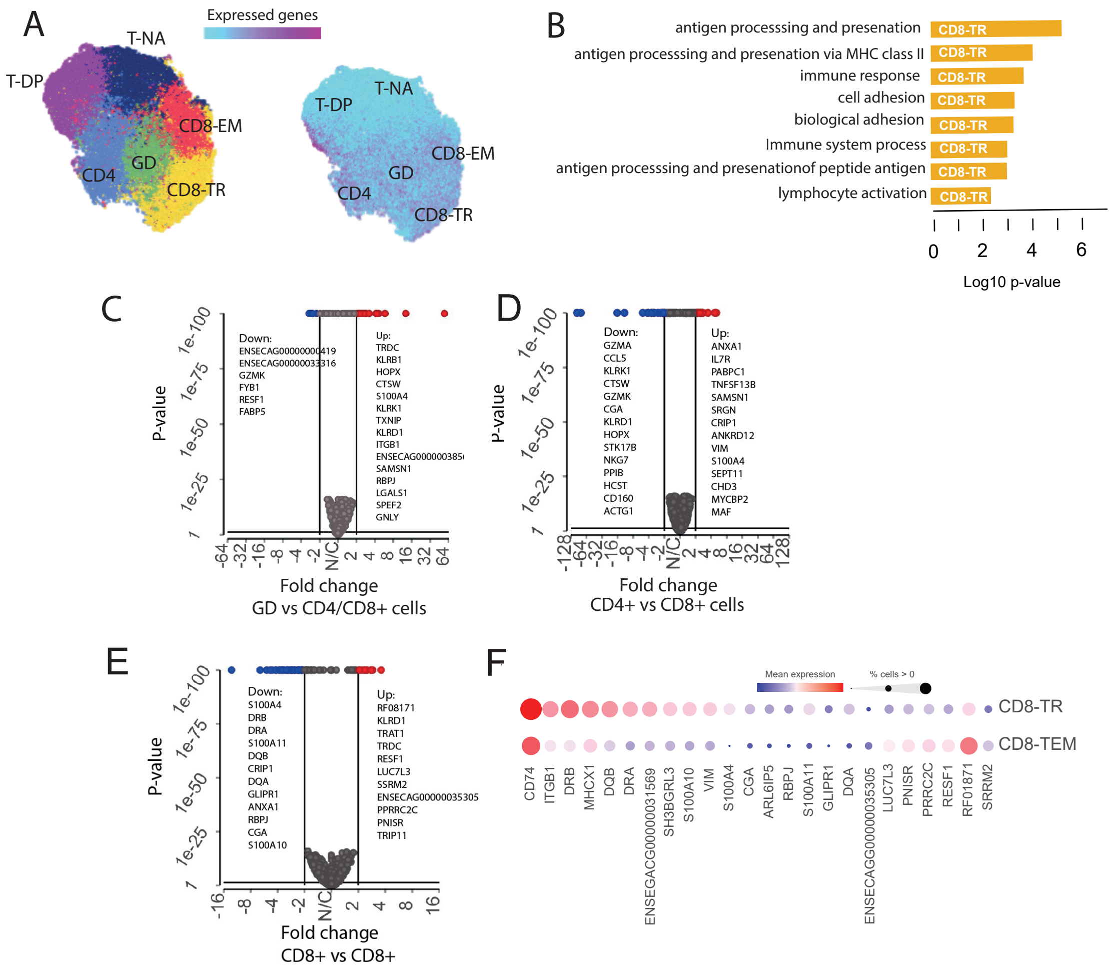


**Figure S4**.
UMAP plot showing the six original T cell clusters (left panel) and the number of genes detected per cell mapped onto the UMAP plot (right panel). The cluster $T_{NA}$ was characterized by a lower number of genes detected and displayed only subtle differential patterns compared to the other T cell clusters. The cluster $T_{DP}$ exhibited slightly increased expression of alveolar macrophage markers compared to the other T-cell clusters.
B) Gene ontology pathways upregulated in the $CD8^{+}_{TR}$ cluster.
C) Volcano plots showing differentially expressed gene analysis (MAST) of gamma-delta T cells compared to $CD4^{+}$ and $CD8^{+}$ cells.
D) Volcano plots showing differentially expressed gene analysis analysis (MAST) of $CD8^{+}_{EM}$ vs $CD8^{+}_{TR}$.
E) Volcano plots showing differentially expressed gene analysis (MAST) of $CD8^{+}_{EM}$ vs $CD8^{+}_{TR.}$
F) Bubble map showing a number of the genes differentially expressed between the two $CD8^{+}$ T cell populations.

| Term name (CD4+ ISG-Hi) | Term ID | $p_{adj}$ | $-\log_{10}(p_{adj})$ |
|---|---|---|---|
| biological process involved in interspecies interaction betwe... | GO:0044419 | $1.157\times10^{-9}$ | |
| response to other organism | GO:0051707 | $3.628\times10^{-9}$ | |
| response to external biotic stimulus | GO:0043207 | $3.691\times10^{-9}$ | |
| response to biotic stimulus | GO:0009607 | $6.254\times10^{-9}$ | |
| defense response to other organism | GO:0098542 | $7.184\times10^{-8}$ | |
| response to virus | GO:0009615 | $8.119\times10^{-8}$ | |
| defense response to virus | GO:0051607 | $1.116\times10^{-7}$ | |
| defense response to symbiont | GO:0140546 | $1.179\times10^{-7}$ | |
| response to cytokine | GO:0034097 | $5.050\times10^{-7}$ | |
| response to external stimulus | GO:0009605 | $1.666\times10^{-6}$ | |
| innate immune response | GO:0045087 | $3.656\times10^{-6}$ | |

| Term name (CD4+ S100-Hi) | Term ID | $p_{adj}$ | $-\log_{10}(p_{adj})$ |
|---|---|---|---|
| actin cytoskeleton organization | GO:0030036 | $3.925\times10^{-7}$ | |
| actin filament-based process | GO:0030029 | $1.306\times10^{-6}$ | |
| cytoskeleton organization | GO:0007010 | $2.177\times10^{-4}$ | |
| supramolecular fiber organization | GO:0097435 | $8.014\times10^{-4}$ | |
| regulation of cell morphogenesis | GO:0022604 | $1.150\times10^{-2}$ | |
| positive regulation of cell adhesion | GO:0045785 | $1.427\times10^{-2}$ | |
| cell junction organization | GO:0034330 | $1.840\times10^{-2}$ | |
| actin filament organization | GO:0007015 | $2.084\times10^{-2}$ | |
| positive regulation of T cell activation | GO:0050870 | $3.805\times10^{-2}$ | |

**Figure S5**. Output from g:Profiler displaying significantly upregulated pathways in CD $4^{+}ISG^{Hi}$ and $CD4^{+}S100^{Hi}$ clusters.

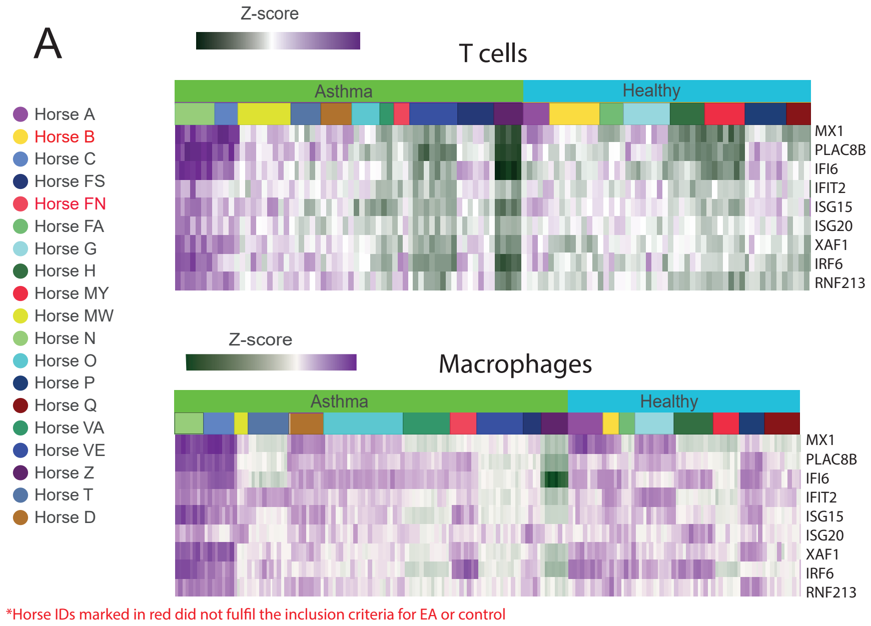


B

| Gene | P-value adj | FC |
|---|---|---|
| PLAC8B | $< 10^{-10}$ | 8.4 |
| MX1 | $< 10^{-10}$ | 7.2 |
| IFI6 | $< 10^{-10}$ | 5.7 |
| XAF1 | $< 10^{-10}$ | 2.5 |
| IRF7 | $< 10^{-10}$ | 2.5 |
| RNF213 | $< 10^{-10}$ | 2.2 |
| ISG15 | $< 10^{-10}$ | 2.2 |

C

| Gene | P-value adj | FC |
|---|---|---|
| PLAC8B | $< 10^{-10}$ | 6.7 |
| MX1 | $< 10^{-10}$ | 9.8 |
| IFI6 | $< 10^{-10}$ | 2.3 |
| XAF1 | $< 10^{-10}$ | 5.1 |
| IRF7 | $< 10^{-10}$ | 4.0 |
| RNF213 | $< 10^{-10}$ | 2.8 |
| ISG15 | $< 10^{-10}$ | 2.8 |

**Figure S6**.
A) Heatmaps showing expression of a subset of ISG genes in individual horses and by group (asthma = green block, healthy= blue block) in T cells and alveolar macrophages, respectively.
B) Differential expression analysis (MAST) shows significant upregulation of a subset of interferon stimulated genes in T cells from two horses (Horses N and C) when tested against the other EA horses.
C) Differential expression analysis (MAST) shows significant upregulation of a subset of interferon stimulated genes in AMs from two horses (Horses N and C) when tested against the other EA horses. These results should, however, be interpreted with caution as the increased expression of certain ISG genes was only observed in a subgroup of two horses.

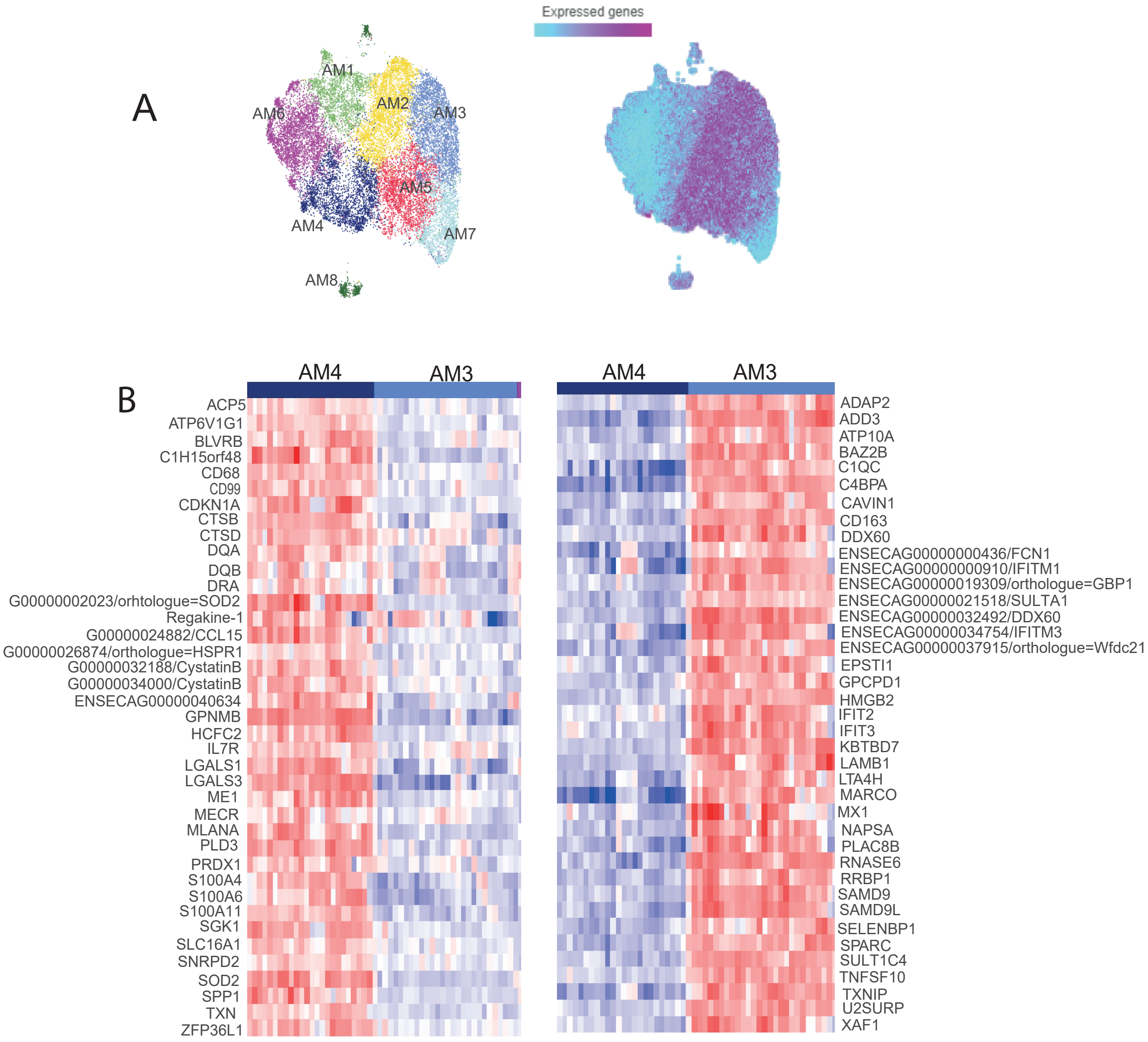


**Figure S7.**
A) The number of detected genes per cell mapped onto the UMAP indicates that the clustering of AMs was partly driven by gene expression levels. Clusters AM6, AM1, AM7 exhibited only subtle gene expression patterns that overlapped with the other populations and could not confidently be annotated. Cluster AM8 mainly displayed increased expression of ribosomal protein genes.
B) Heatmap showing top differentially expressed (upregulated) genes in AM4 vs AM 3 cluster (left panel) and AM3 vs AM4 cluster (right panel).

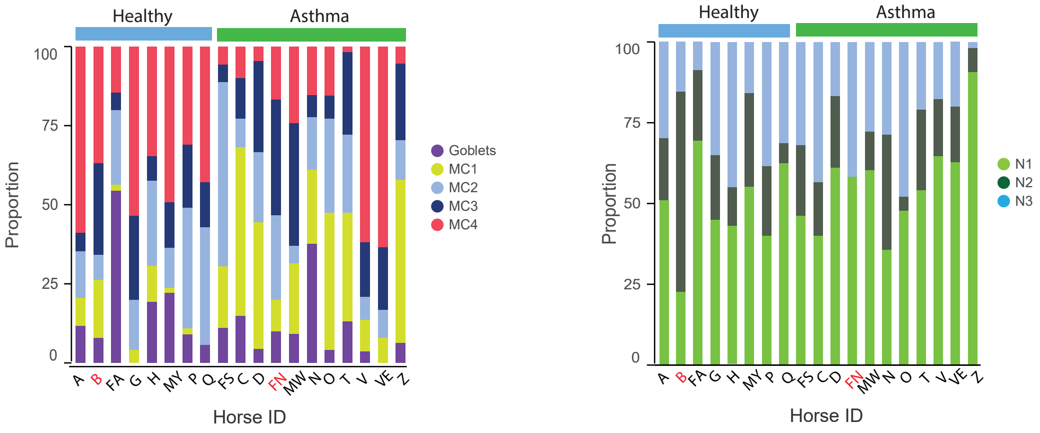


*Horse IDs marked in red did not fulfil the inclusion criteria for EA/control

**Figure S8.** Mast cell cluster proportions in individual horses (left panel). Neutrophil cluster proportions in individual horses (right panel).

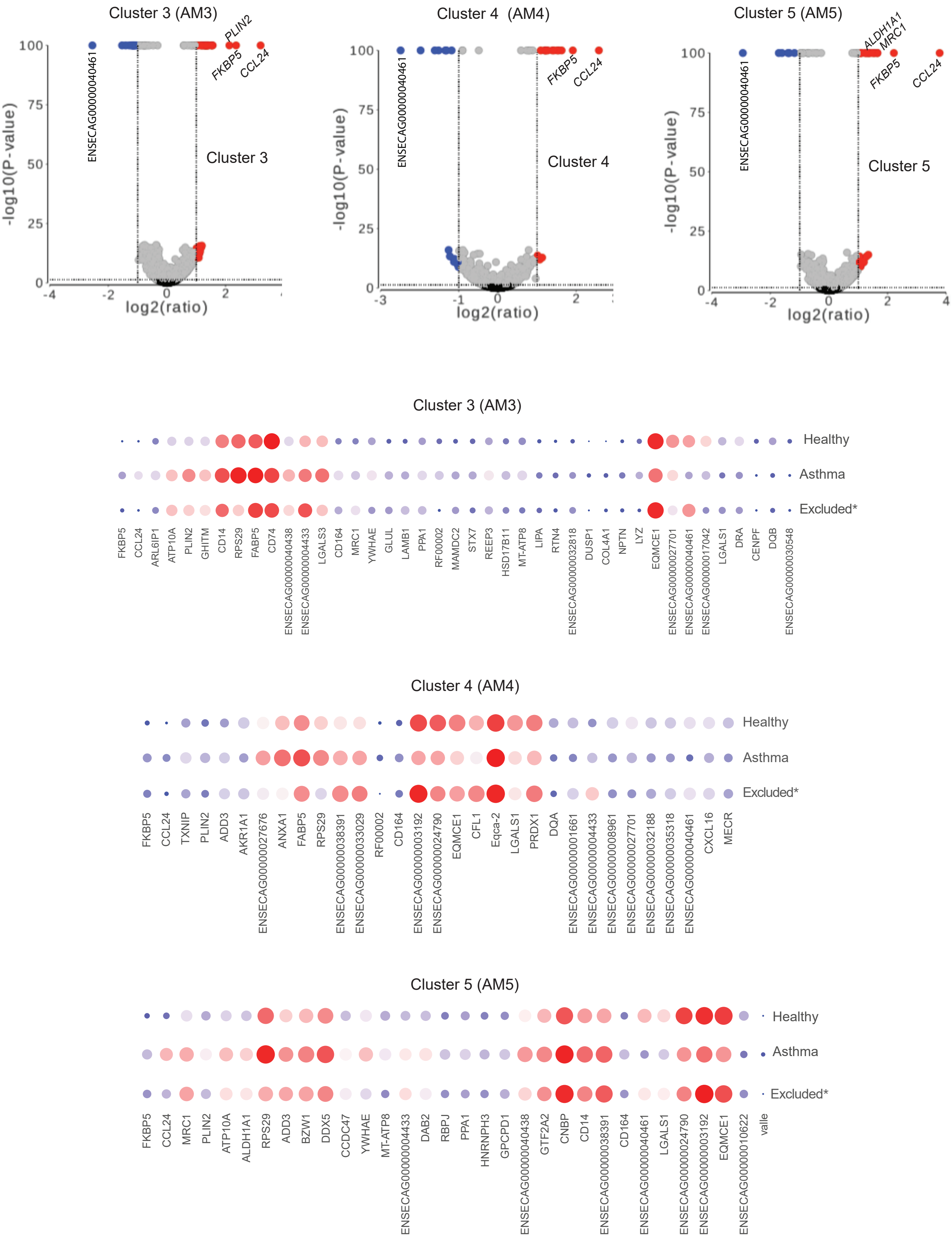


**Figure S9.**
Differential expressed gene analysis (MAST) between asthma and healthy horses (all horses included), performed cluster-wise in alveolar macrophage subpopulations AM3, AM4 and AM5. The results are visualized by volcano plots (top row) and bubble plots. *Exluded = Horses B and FN

| **Genes up in asthma horses** | **Pseudo bulk DE FC >2, FDR <0.1** | **Single cell DE FC >2, FDR <0.1** |
|---|---|---|
| FKBP5 | AM, T-cells, Mast cells | AM, T-cells, Mast cells |
| CCL24 | AM, T-cells, | AM |
| RGS1 | mast cells | mast cells |
| RGS13 | | mast cells |
| TXNIP | | AM4, mast cells, neutrophils |
| PLIN2 | AM | AM3, AM4, AM5 |
| ATP10A | n.d | AM |
| PECAM1 | AM | n.d |
| ENSECAG00000034004/O=FCGER1A | AM | n.d |
| GOS2 | AM | n.d |
| DUSP1 | AM | neutrophils |
| CD164 | | AM3, AM4 |
| GLUL | n.d | AM |
| LAMB1 | | AM3 |
| ANXA1 | | AM4 |
| MRC1 | | AM5 |
| ALDH1A1 | | AM5 |
| ENSECAG00000038391/Eqca-1 | mast cells | T-cells, mast cells, neutrophils, AM5 |
| ENSECAG00000036180/novel gene | mast cells | mast cells |
| EPB41L2 | n.d | mast cells |
| ENSECAG00000028889/MT | AM | n.d |
| EVI2B | n.d | neutrophils |

n.d = not detected (or not significant, FC<2)

## Table S7.

Table S7. Examples of DE genes asthma vs healthy see table S8-S10 for complete lists. For subcluster level DE tests (AM 3,4,5) only MAST was used.

| Primer name | Sequence 5’ -> 3’ |
| --- | --- |
| Macosco TSO | AAGCAGTGGTATCAACGCAGAGTGAATrGrGrG |
| New P5 SMART PCR Hybrid | AATGATACGGCGACCACCGAGATCTACACGCCTGTCCGCGGAAGCAGTGGTATCAACGCAGAGT*A*C |
| SMART PCR | AAGCAGTGGTATCAACGCAGAGT |
| Nextera N7XX | CAAGCAGAAGACGGCATACGAGATTCGCCTTAGTCTCGTGGGCTCGG |
| Read1CustomSeqB | 5’GCCTGTCCGCGGAAGCAGTGGTATCAACGCAGAGTAC |

**Supplementary Table 2.** Primer Sequences
