# Supplementary material for: Single-cell transcriptomics delineates the immune cell landscape in equine lower airways and reveals upregulation of FKBP5 in horses with asthma
Source: Sci Rep. 2023 Sep 27;13:16261. doi: 10.1038/s41598-023-43368-4 (PMC10533524; doi:10.1038/s41598-023-43368-4)
Supplement: Supplementary file 10 — Supplementary Table S11. [file 41598_2023_43368_MOESM10_ESM.pdf]

Primer name	Sequence 5’ -> 3’
Macosco TSO	AAGCAGTGGTATCAACGCAGAGTG AATrGrGrG
New P5 SMART PCR Hybrid	AATGATACGGCGACCACCGAGATC TACACGCCTGTCCGCGGAAGCAGT GGTATCAACGCAGAGT*A*C
SMART PCR	AAGCAGTGGTATCAACGCAGAGT
Nextera N7XX	CAAGCAGAAGACGGCATACGAGAT TCGCCTTAGTCTCGTGGGCTCGG
Read1CustomSeqB	5’GCCTGTCCGCGGAAGCAGTGGTA TCAACGCAGAGTAC

Table S11
